# Cav3.1-driven bursting firing in ventromedial hypothalamic neurons exerts dual control of anxiety-like behavior and energy expenditure

**DOI:** 10.1038/s41380-022-01513-x

**Published:** 2022-03-22

**Authors:** Jie Shao, Da-Shuang Gao, Yun-Hui Liu, Shan-Ping Chen, Nian Liu, Lu Zhang, Xin-Yi Zhou, Qian Xiao, Li-Ping Wang, Hai-Lan Hu, Fan Yang

**Affiliations:** 1 The Brain Cognition and Brain Disease Institute, Shenzhen Institute of Advanced Technology, Chinese Academy of Sciences, Shenzhen-Hong Kong Institute of Brain Science-Shenzhen Fundamental Research Institutions, Shenzhen, 518055 China; 2 University of Chinese Academy of Sciences, Beijing, 100049 China; 3 Department of Psychiatry of First Affiliated Hospital, Zhejiang University School of Medicine, Hangzhou, 310058 China

**Keywords:** Neuroscience, Psychology

## Abstract

The central nervous system has evolved to coordinate the regulation of both the behavior response to the external environment and homeostasis of energy expenditure. Recent studies have indicated the dorsomedial ventromedial hypothalamus (dmVMH) as an important hub that regulates both innate behavior and energy homeostasis for coping stress. However, how dmVMH neurons control neuronal firing pattern to regulate chronic stress-induced anxiety and energy expenditure remains poorly understood. Here, we found enhanced neuronal activity in VMH after chronic stress, which is mainly induced by increased proportion of burst firing neurons. This enhancement of VMH burst firing is predominantly mediated by Cav3.1 expression. Optogenetically evoked burst firing of dmVMH neurons induced anxiety-like behavior, shifted the respiratory exchange ratio toward fat oxidation, and decreased food intake, while knockdown of Cav3.1 in the dmVMH had the opposite effects, suggested that Cav 3.1 as a crucial regulator. Interestingly, we found that fluoxetine (anxiolytics) could block the increase of Cav3.1 expression to inhibit the burst firing, and then rescued the anxiety-like behaviors and energy expenditure changes. Collectively, our study first revealed an important role of Cav3.1-driven bursting firing of dmVMH neurons in the control of anxiety-like behavior and energy expenditure, and provided potential therapeutic targets for treating the chronic stress-induced emotional malfunction and metabolism disorders.

## Introduction

During the evolution, the exposure to stressors exert persistent pressure on the central nervous system to regulate both the homeostasis of energy expenditure and the innate behavior response to the environment [1–3]. Such demands for stress coping and survival have also facilitated the coevolution of the metabolic regulation and innate behavior response within the identical brain nucleus [2, 4]. On the other hand, long-term stress could affect normal neural activities in specific brain nucleus [5, 6], to induce both anxiety-like behavior [7, 8] and glucose or lipid metabolism disorders [3, 4, 9, 10]. Recently, many efforts have been expended to identify the crucial central nodes that interconnect the regulation of emotion and metabolic processes [3, 9, 11, 12]. The hypothalamus, especially the ventromedial hypothalamus (VMH), has been found to play crucial roles in controlling both innate survival behaviors [13, 14] and energy homeostasis [12, 15, 16].

The VMH is an evolutionarily conserved deep subcortical nucleus [17]. While the ventrolateral part (vl) of the VMH modulates a series of social behaviors [18–20], the dorsomedial part (dm) is specifically involved in maintaining energy homeostasis [15, 16, 21, 22] and stress response [14, 23]. Oscillations in the dmVMH is affected by metabolism-related peptides and can act on sympathetic excitation [24, 25]. Steroidogenic-factor-1 (SF-1)-expressing neurons are enriched in the dmVMH and play important roles in controlling food intake and energy expenditure [15, 16, 21, 22]. Optogenetic activation of VMH SF-1 neurons induces aversive behavior in mice [14, 23], and it has implicated that a complex neuronal networks exists among VMH SF-1 neurons to process aversive stimulus [26]. Given the complicated functions of SF-1 neurons [20], it is important to dissect how molecular or electrophysiological distinct neuronal subtypes in dmVMH regulate emotional state and energy homeostasis.

Recent studies have demonstrated the important role of burst firing neurons in regulating emotional state and metabolic functions; neuronal burst firing can promote oscillation and is critical for the transmission of information and regulation of specific physiological processes in crucial brain areas [27–32]. In the hippocampus, burst firing of CA1 pyramidal neurons play an important role in regulating N-methyl-d-aspartate (NMDA)-mediated transmission and the epileptogenic mechanism [27], and the bursting output of CA1 (sharp wave ripples) has been implicated in the regulation of peripheral glucose homeostasis [33]. In the lateral habenula, NMDA-receptor-and-T-VGCC-dependent bursting activity are increased after chronic stress and, most strikingly, can drive depression-like behaviors [30]. In the ventral subiculum, chronic social defeat stress increases both T-VGCC-mediated currents and expression of Cav3.1 protein [31]. All these evidence suggest that bursting firing may be involved in the anxiety-like behavior changes during the stress coping. However up to now, the specific function of burst firing in dmVMH during the stress is elusive, how the dmVMH neurons control burst firing pattern to regulate anxiety and related energy expenditure changes is not clear.

In this study, we established a chronic-stress mouse model that induced anxiety-like behavior and decreased energy expenditure. We found a significantly increased proportion of bursting neurons under chronic stress conditions, which was mainly caused by elevated T-type calcium channel Cav3.1 expression. This enhancement of burst firing contributes to the altered neural activity in VMH after chronic stress. Importantly, optogenetically induced dmVMH burst firing drive anxiety-like behaviors, shifted respiratory exchange ratio (RER) and decreased average energy expenditure in naïve mice. Conversely, knockdown of Cav3.1 or administration of fluoxetine rescued anxiety-like behavior and energy expenditure changes through inhibition of the burst firing in the dmVMH. Taken together, we are the first to identify Cav3.1-mediated burst firing pattern changes in the dmVMH during the chronic stress, and also demonstrate its crucial role in regulating both anxiety-like behavior and energy expenditure changes.

## Materials and methods

Animals, chronic stress procedures, behavioral tests, CLAMS (comprehensive laboratory animal monitoring system) tests, electrophysiology recording, immunostaining, ELISA (enzyme linked immunosorbent assay), RNA isolation and PCR, photometry recording, stereotaxic surgery and viral injection, optogenetics manipulation, and statistical analysis were performed as described in the Supplementary materials and methods section.

## Results

### Chronic stress-induced anxiety-like behavior and related energy expenditure change

**Fig. 1:**
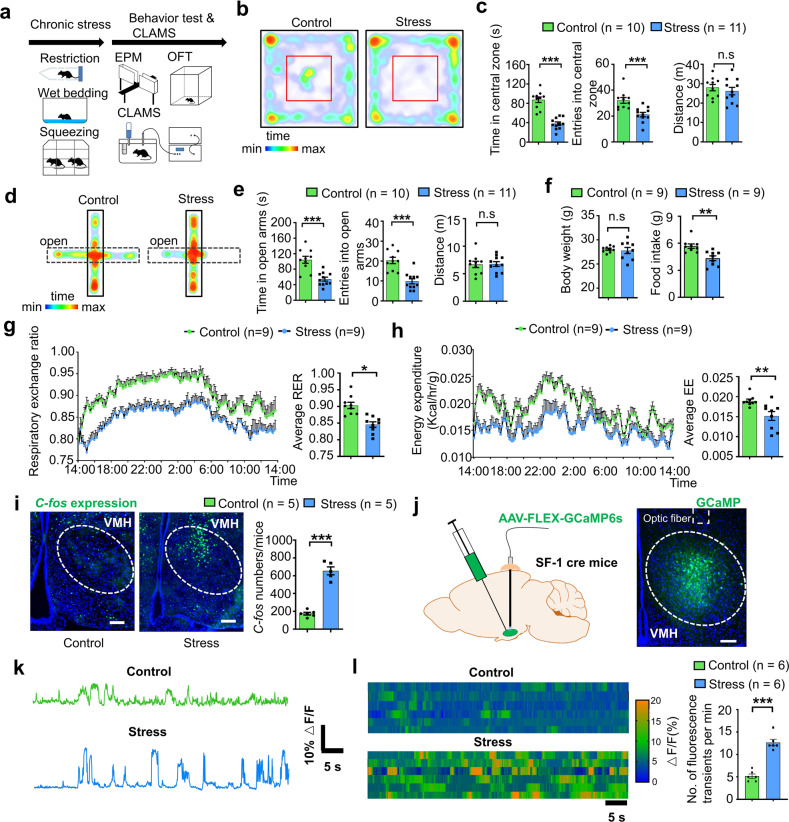
Stressed mice exhibited anxiety-like behavior, altered metabolism, and enhanced neural activity in dmVMH. **a** Illustration of unpredictable chronic stress protocol and phenotype assessment, with one stressor treatment randomly chosen per day, lasting for 4 weeks. **b** Residence time in each site of open field (blue, less time; red, more time). **c** Behavioral analysis of control (*n* = 10) and stress group mice (*n* = 11) in open field test showed significant decrease in both time spent in central area (unpaired Student’s *t*-test, *P* < 0.0001) and entries into central area in stress group (unpaired Student’s *t*-test, *P* = 0.0009), but no obvious change in traveling distance between groups (unpaired Student’s *t*-test, *P* = 0.4896). **d** Residence time in each site of elevated plus-maze (blue, less time; red, more time) of control and stress groups. **e** Control (*n* = 10) and stress groups (*n* = 11) in elevated plus-maze showed significant decrease in time spent in open arm (unpaired Student’s *t*-test, *P* < 0.0001) and entries into open arm in stress group (unpaired Student’s *t*-test, *P* < 0.0001), but no obvious change in traveling distance between groups (unpaired Student’s *t*-test, *P* = 0.9215). **f** Body weight monitored after chronic stress period showed no obvious change compared with control group (*n* = 9 in each group, unpaired Student’s *t*-test, *P* = 0.7125), and 24-h food intake after overnight fasting decreased in stress group (*n* = 9, unpaired Student’s *t*-test, *P* = 0.0014) compared with control group (*n* = 9). **g** Average respiration exchange ratio (RER) decreased in stress group compared with control group (unpaired student’s *t*-test, *P* = 0.0003, *n* = 9 mice in each group); RER curve shifted after chronic stress (two-way ANOVA, *P* = 0.0002, *F* (1, 16) = 21.95, with Bonferroni correction). **h** Significant decreases in 24-h energy expenditure (EE) curve and average EE were observed in stress group (two-way ANOVA, *P* = 0.0057, *F* (1, 16) = 20.2, with Bonferroni correction; unpaired Student’s *t*-test; *P* = 0.0060). **i** Increased *c-fos* expression in dmVMH under chronic stress (unpaired Student’s *t*-test, *P* < 0.001, *n* = 5 mice in each group). Scale bar, 100 µm. **j** Schematic of GCaMP6s injection and expression in VMH SF-1 neuron. Scale bar, 100 µm. **k**, **l** Representative in vivo calcium fluorescence traces and heat maps demonstrate enhanced spontaneous calcium signals (dF/F) of SF-1 neurons in mice of stress group compared with control (6 mice in each group, unpaired Student’s *t*-test, *P* < 0.001). Data are means ± SEM. *P < 0.05, **P < 0.01, ***P < 0.001. CLAMS comprehensive laboratory animal monitoring system.

Repeated exposure to stressors modifies the activity of neuronal circuits associated with a variety of abnormalities, including behavior, emotional state, and energy expenditure [2], which adaptively or maladaptively respond to the stressful situation. To probe the impact of chronic stress on behavior and energy metabolism, we applied unpredictable chronic stressors to establish a stress mouse model. During the chronic stress period, mice were exposed to random stressors for 4 weeks, as illustrated in Fig. 1a, with no stressors used in the control group. Both the control and stress groups were tested in open field test (OFT) and elevated plus maze test (EPM). Consistent with previous reports [7, 8], we found that chronic stress-induced a state of anxiety (Fig. 1b–e). Compared with the control group, mice in the stress group spent less time in the central area of the open field and open arm of the EPM without any change in locomotion distance (Fig. 1c–e). The number of entries into the central area and open arms also decreased in the stress group compared with the control group (Fig. 1c–e).

We next explored whether chronic stress affected energy expenditure in stressed mice. The body weight of each mouse was measured after the chronic stress period, and no significant difference was observed between the control and stress groups (Fig. 1f). Both the RER and food intake were monitored for 24 h using CLAMS cages. Results showed that the average RER of the stress group was much lower than that of the control group, which represented a shift toward fat oxidation (Fig. 1g). Energy expenditure was calculated from oxygen consumption and RER. Average energy expenditure also showed a significant decrease in stressed mice (Fig. 1h). To determine the underlying cause of different RER in the treatment groups, we also analyzed food intake and found that caloric intake in stressed mice was significantly lower than that in the controls (Fig. 1f). Taken together, these findings suggest that chronic stress can induce anxiety-like behavior, reduce food consumption, and shift the RER toward fat oxidation.

Given its important role in regulating anxiety, feeding, and energy expenditure [2, 22], we hypothesized that the dmVMH may be involved in the above chronic stress-induced changes. After behavioral tests, mice were perfused, and *c-fos* expression was determined via immunostaining. Higher *c-fos* expression was found in the dmVMH of chronically stressed mice than in that of the control group (Fig. 1i), suggesting that more dmVMH neurons were activated under chronic stress conditions. We also employed in vivo photometry to record spontaneous calcium activity of VMH SF-1 neurons. Mice were injected with GCamMP6s AAV vector and implanted with optic fiber (Fig. 1j) after chronic stress. Compared with control group, mice in stress group demonstrated obvious increase of spontaneous calcium signal (both of amplitude and frequency) in dmVMH (Fig. 1k, l). However, calcium signals did not differ significantly before and after entry into the central region of the OFT or open arms of the EPM in either the control or stress groups (Supplementary Fig. 1). Moreover, result of in vivo recording of local field potential in dmVMH also demonstrated that stressed mice exhibited higher theta band power than that of control mice (Supplementary Fig. 2). Together with the *c-fos* staining results, our data suggest that dmVMH neuronal activity is significantly changed after long-term repeated stress, and might represent an emotion state of anxiety.

### Chronic stressors induced burst firing in dmVMH

**Fig. 2:**
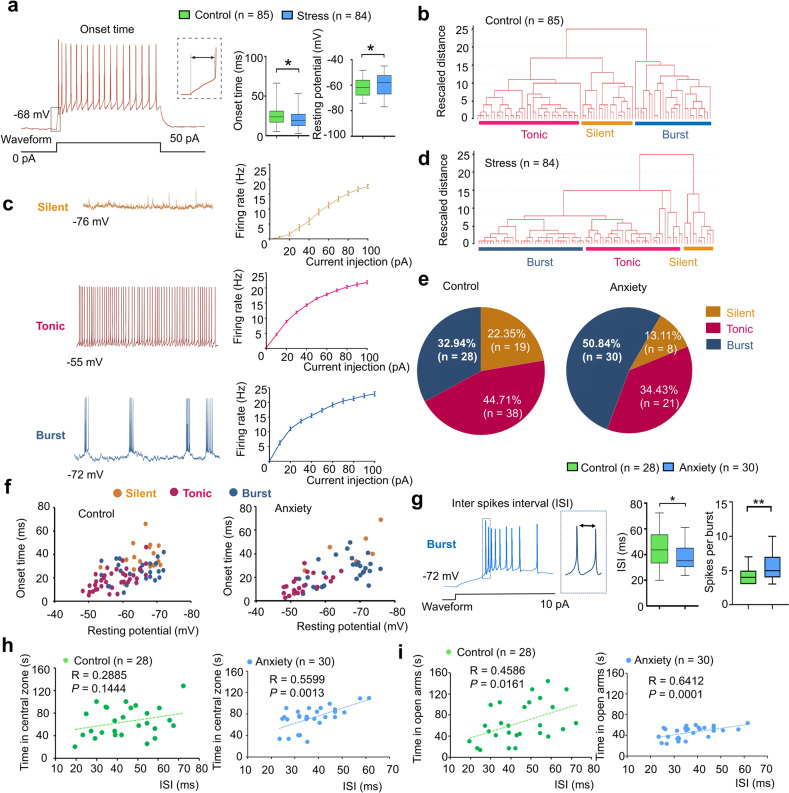
Chronic stress-induced enhancement of burst firing in dmVMH neurons. **a** Decreased average onset time (unpaired Student’s *t*-test, *P* = 0.0233) and depolarized average resting membrane potential (RMP) (unpaired Student’s *t*-test, *P* = 0.0434) in 84 dmVMH neurons from stressed mice compared with 85 neurons from wild-type control mice. **b** Cluster analysis of 85 dmVMH neurons from 35 control mice. Dendrogram of cluster analysis shows that dmVMH neurons could be classified into three subtypes: i.e., silent, tonic-firing, and bursting. **c** Electrophysiological properties of silent, tonic-firing, and bursting dmVMH neuronal subtypes. left: whole-cell recording traces of three neuronal subtypes without current injection; right: frequency-current curve of three subtypes at current injections of 0–100 pA and 10 pA/step. **d** Cluster analysis of 84 dmVMH neurons from 39 stressed mice. Dendrogram of cluster analysis shows these dmVMH neurons can be classified into three subtypes: i.e., silent (*n* = 11), tonic-firing (*n* = 35), and bursting (*n* = 38). **e** Pie chart of percentages of neuronal dmVMH subtypes in control group and stressed mice with obvious anxiety-like behavior (anxiety group). **f** Distribution of 85 dmVMH neurons in control mice (left) and 57 dmVMH neurons in anxiety group (right) using onset time-RMP coordinate system, which represents shorter average onset time and more depolarized average RMP caused by changes in proportion of three subtypes. **g** Inter-spike interval (ISI) of bursts in dmVMH neurons of control and stressed mice. Left, Example of burst firing and ISI; right, ISI of burst firing dmVMH neurons (*n* = 30) in anxiety group decreased significantly compared with that in control group (*n* = 28), and burst of anxiety group exhibited more spikes (unpaired Student’s *t*-test, ISI: *P* = 0.0173, spikes in each burst, *P* = 0.0036). **h**, **i** ISI of dmVMH bursting neurons in anxiety group (stressed mice which displayed obvious anxiety behavior) exhibits higher correlation with the residing time in open arms of EPM and central area of open field, compared with that in control group (control group: *n* = 28 cells from 21 mice; anxiety group: *n* = 30 cells from 20 mice). The box plotted at the median extending from the 25 to 75th percentile, and the whisker represents Min to Max distribution. Data are means ± SEM *P < 0.05, **P < 0.01.

The above experiments revealed enhanced neuronal activity in the dmVMH under chronic stress, but the underlying mechanism was unclear. To explore which electrophysiological characteristics of dmVMH neurons changed after chronic stress, we recorded 84 dmVMH neurons from 38 stressed mice and 85 dmVMH neurons from 32 control mice under whole-cell patch clamp configuration and analyzed the electrophysiological characteristics. Our data demonstrated that these neurons displayed shorter onset time and depolarized RMP, on average, compared with the control group (Fig. 2a). The alterations implied that the overall excitability of dmVMH neurons was enhanced under chronic stress, consistent with the *c-fos* immunostaining and in vivo photometry results (Fig. 1i–l).

We carried out a comprehensive analysis of the membrane properties of dmVMH neurons to quantitatively determine their electrophysiological diversity (Fig. 2b). To classify these neurons, we measured and analyzed eight electrophysiological parameters (Supplementary Table 1) and performed cluster analysis. The resulting dendrogram in Fig. 2b (with rescaled distance shown along the vertical axis) illustrates the similarity between clusters in control group. Our analysis showed that all neurons could be divided into three subtypes: i.e., silent, tonic-firing, bursting. These three dmVMH neuronal subtypes displayed distinct electrophysiological properties (Fig. 2c, Supplementary Fig. 3).

We then applied cluster analysis using the same methods as depicted above (Fig. 2d) to explore overall changes in the electrophysiological properties of dmVMH neurons in stress group. We found that dmVMH neurons in stressed mice could also be divided into three subtypes. Onset time, RMP and recording site of these three subtypes were also compared with those of the control group (Supplementary Fig. 4). By analyzing data from behavior tests and electrophysiological recordings, we found the proportion of burst firing neurons in stress group, especially in mice with obvious anxiety-like behavior (anxiety group), increased significantly compared with that in the control group, whereas the proportion of the other subtypes decreased (Fig. 2d, e). This alteration could explain why dmVMH neurons from stressed mice demonstrated a shorter average onset time of action potentials induced by 50 pA current injection (Fig. 2a), as burst firing neurons displayed a similar onset time as tonic-firing neurons but a shorter onset time compared with silent neurons (Fig. 2f, Supplementary Fig. 4). In addition, the increased proportion of bursting neurons and more depolarized tonic-firing neurons (Fig. 2f, Supplementary Fig. 4) may have contributed to the higher average RMP in dmVMH neurons after chronic stress (Fig. 2a). Furthermore, average inter-spike interval (ISI, between first and second spike of a burst) in the dmVMH burst firing neurons was shorter in the anxiety group than in the control group, consistent with the greater number of spikes firing in a single burst (Fig. 2g). We also found that ISI of dmVMH bursting neurons was more correlated with the time in central area or open arms in mice of anxiety compared with the mice in control group (Fig. 2h, i). Taken together, these results consistently supported the relationship between increased bursting activity in the dmVMH and chronic stress-induced anxiety.

### Optogenetic manipulation of burst firing neurons in dmVMH induced anxiety-like behavior and energy expenditure change

**Fig. 3:**
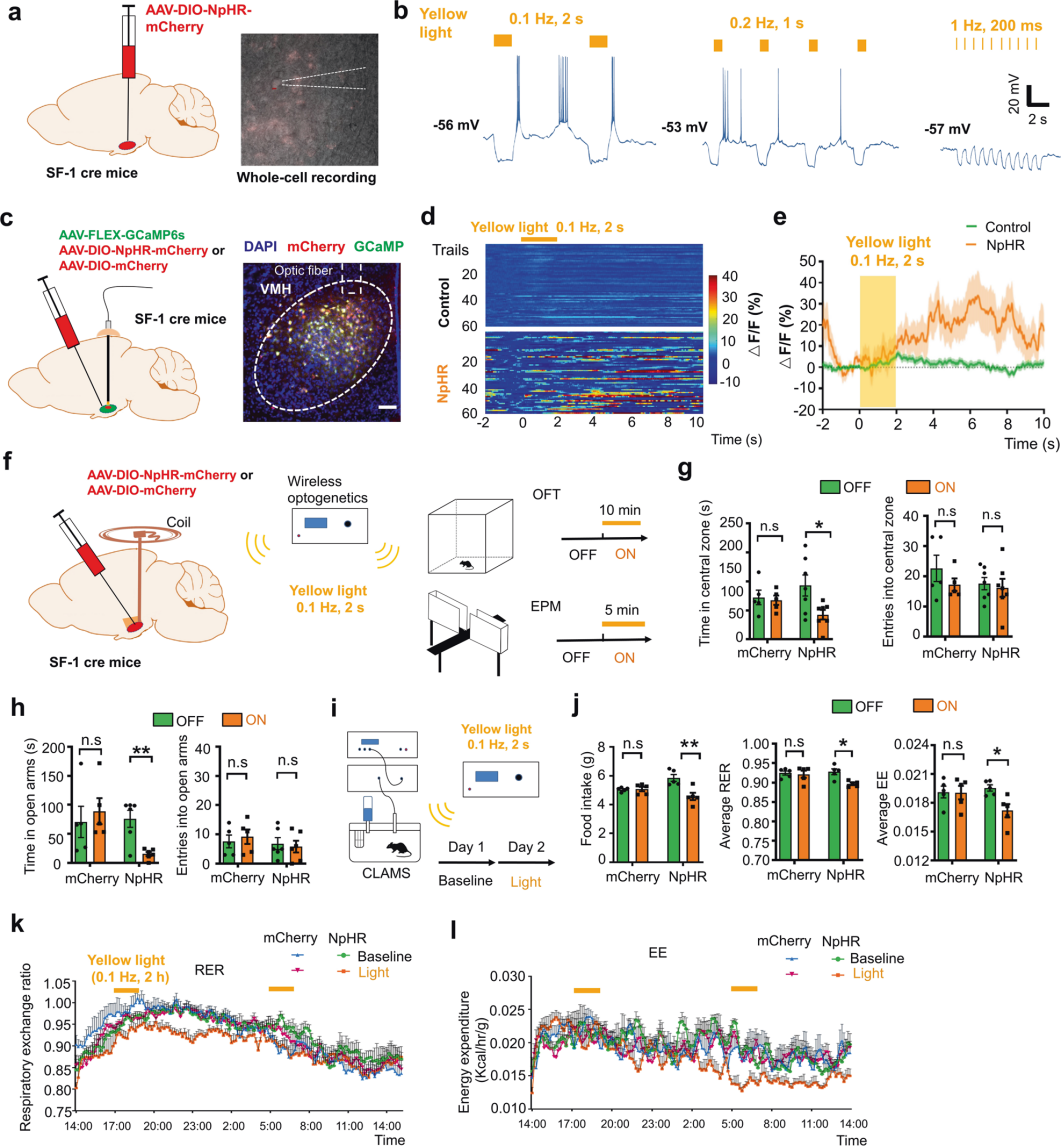
Optogenetic activation of burst firing neurons in dmVMH induced anxiety-like behavior and energy expenditure changes. **a** Schematic of dmVMH injection of NpHR AAV viral vector to induce burst firing in vivo. **b** Whole-cell recordings of yellow light-evoked burst firing in brain slices (yellow light: 590 nm, left: 0.1 Hz, 2 s; middle: 0.2 Hz, 1 s; right: 1 Hz, 200 ms), 0.1 Hz successfully induced activation of burst firing neurons in dmVMH. **c** Schematic of dmVMH injection of NpHR AAV and GCaMP6s AAV viral vector in SF-1 cre mice to achieve in vivo photometry recording and optogenetic manipulation. Scale bar, 100 µm. **d** Heatmaps demonstrate GCaMP6s fluorescence change induced by optogenetically induced burst firing (60 trails from 3 mice in each group). **e** Average traces of calcium fluorescence responses in VMH evoked by yellow light. **f** Illustration of wireless optogenetic manipulation of dmVMH neurons and behavioral test strategies in free-moving mice. **g** Open field tests before and during light illumination: residence time in central area decreased during 10-min yellow light illumination in NpHR group (*n* = 7, paired Student’s *t*-test, *P* = 0.0210), but not control group (*n* = 5, paired Student’s *t*-test, *P* = 0.4587). No significant effect of light stimulation was observed on number of entries into central area in two groups (paired Student’s *t*-test, control group: *P* = 0.3915; NpHR group: *P* = 0.5327). **h** Residence time in open arms decreased only in NpHR group (paired Student’s *t*-test, control group: *n* = 5, *P* = 0.6382; NpHR group: *n* = 6, *P* = 0.0036); No obvious changes in number of entries into open arms observed during “light-on” period in two groups (paired Student’s *t*-test, control group: *P* = 0.4908; NpHR group: *P* = 0.7060). **i** Schematic of CLAMS experiments on free-moving mice with wireless optogenetic manipulation of dmVMH neurons, 0.1-Hz yellow light illumination at the start of the test period at Day 2 for 2 h and repeated the stimulation after 12 h. **j** Food intake and energy expenditure were monitored during optogenetic manipulation of dmVMH neurons in free-moving mice. Food intake decreased during light stimulation in NpHR group (paired Student’s *t*-test, control group: *P* = 0.6240; NpHR group: *P* = 0.0038, *n* = 5 mice in each group), also average RER (paired Student’s *t*-test, control group: *P* = 0.7058; NpHR group: *P* = 0.0176) and EE (paired Student’s *t*-test, control group: *P* = 0.9502; NpHR group: *P* = 0.0383) decreased in NpHR group. **k**–**l** 24-h-RER and EE curve of NpHR group in Day 2 (0.1 Hz, 2 s yellow light; lasting for 2 h/trial, 2 trials) shifted compare with baseline. (two-way ANOVA, RER: *P* = 0.0053, *F* (1, 8) = 14.38; EE: *P* = 0.0143, *F* (1, 8) = 9.699, with Bonferroni correction), and no significant change occurs in control group (two-way ANOVA, RER: *P* = 0.7709, *F* (1, 8) = 0.0907; EE: *P* = 0.9457, *F* (1, 8) = 0.0049, with Bonferroni correction). Data are means ± SEM; *P < 0.05, **P < 0.01.

Given the enhancement of burst firing in the dmVMH after chronic stress, we investigated whether induced burst firing of dmVMH neurons alone in naïve mice was sufficient to produce a similar response in behavior and energy metabolism. We first applied optogenetic manipulation to elicit a low-threshold spike and mimic the generation of bursting in dmVMH neurons (Fig. 3a). In vitro patch clamp experiments on brain slices indicated that yellow light illumination at 0.1 Hz could slightly hyperpolarize and depolarize bursting neurons periodically, which enabled the re-activation of calcium channels and generation of burst firing during the light “ON-OFF” intervals in bursting neurons (Fig. 3b). The evoked burst firing did not follow the “ON-OFF” intervals when the illumination frequency was over 0.1 Hz (Fig. 3b). In addition, illumination at 0.1 Hz exerted no significant effects on the tonic-firing or silent neuronal subtypes (Supplementary Fig. 5). To investigate whether yellow light illumination could induce VMH SF-1 neurons firing in vivo, we injected with GCamMP6s AAV and NpHR AAV vectors into the VMH of SF-1 cre mice and recorded the calcium activity induced by 0.1 Hz illumination after 4-weeks of expression. Data indicated that a significant increase of calcium signal follows with the yellow light illumination in NpHR-expressing mice (Fig. 3c–e), and this calcium activity lasted for a longer time than the burst firing induced in single neuron in vitro. This result suggests that there might be a complex network among VMH SF-1 neuron to underlies the persistent effect caused by optogenetically induced burst firing, which also supported the findings in previous study [26].

We then applied yellow light illumination to induce burst firing in vivo and performed both behavioral and energy metabolic tests in mCherry or NpHR-expressing mice (Fig. 3f). Light stimulation in NpHR group, but not mCherry group, led to decreased residing time in the central area of the open field and open arm of the EPM compare with “light-off” period, which mimicked the effects of chronic stress-induced anxiety-like behavior (Fig. 3g, h). We also applied wireless optogenetics to induce burst firing of neurons and simultaneously monitored the metabolism of mice using CLAMS (Fig. 3i). We applied 0.1-Hz yellow light illumination at the start of the test period for 2 h and repeated the stimulation after 12 h, with consecutive monitoring of mice energy metabolism for 24 h. Results of NpHR group indicated that average RER, energy expenditure, and food intake in the test period (Day 2) decreased significantly compared with the baseline level before the test period (Fig. 3j–l), while no obvious change observed in control group. Taken together, our results indicated that enhancement of burst firing induced by local optogenetic manipulation in the dmVMH was sufficient to affect anxiety-like behavior and energy expenditure changes, which simulated the phenotypes of chronically stressed mice.

### Burst firing in dmVMH is mediated by T-VGCC

**Fig. 4:**
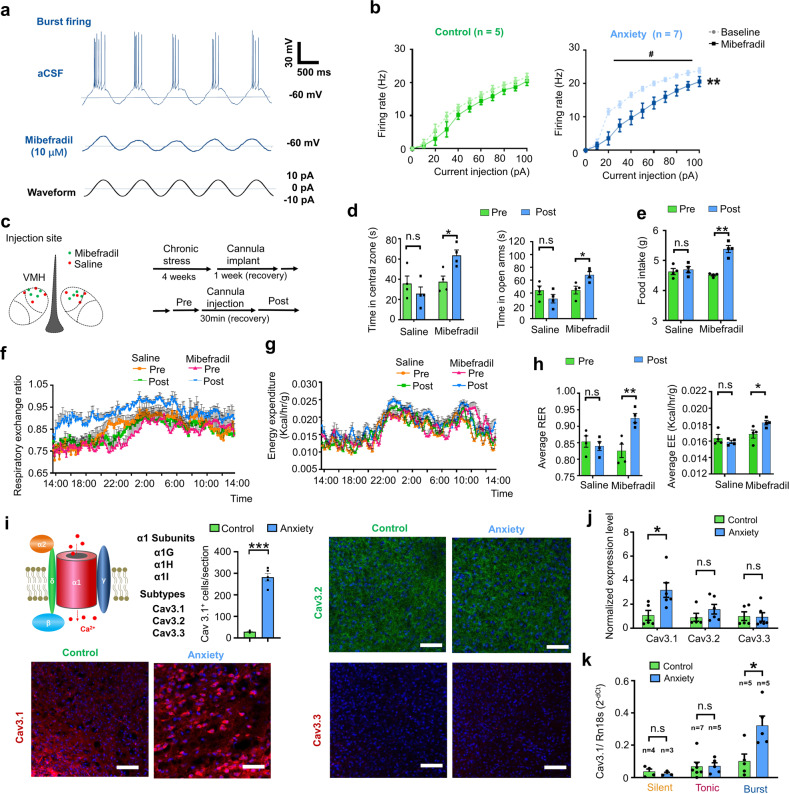
T-VGCC mediated enhancement of burst firing in dmVMH neurons under chronic stress. **a** Evoked burst firing trace of dmVMH neurons without and with T-VGCC antagonist (mibefradil, 10 μM), 10 pA current injection was given in cosine waveform. **b** Effects of mibefradil on suprathreshold activity in dmVMH burst firing neurons in control (*n* = 5 cells from 4 mice) and anxiety groups (*n* = 7 cells from 5 mice). Mibefradil inhibited T-VGCC and caused a rightward frequency-current curve shift (two-way ANOVA, control, *P* = 0.2140, *F* (1, 8) = 1.854; anxiety, *P* = 0.0077, *F* (1, 12) = 10.22, with Bonferroni correction). **c** Schematic of cannula implanted sites and experimental strategy of T-VGCC blockage in the VMH of chronic stress-treated mice. **d** Behavioral test before and after delivery of mibefradil or saline; left, residence time in central area of open field increased after mibefradil application (*n* = 4 in each group, *P* = 0.0142, paired Student’s *t*-test); right, residence time in open arm increased after mibefradil application (*n* = 4 in each group, *P* = 0.0228, paired Student’s *t-*test). **e**–**h** Administration of mibefradil in stressed mice increased food intake (*n* = 4 in each group, *P* = 0.0030, unpaired Student’s *t*-test), average RER (*P* = 0.00671, paired Student’s *t*-test) and average EE (*P* = 0.0482, paired Student’s *t*-test). RER and EE curve shifted (two-way ANOVA, RER: *P* = 0.0067, *F* (1, 6) = 16.42; EE: *P* = 0.0301, *F* (1, 6) = 7.993, with Bonferroni correction) after applying mibefradil. No comparable changes were observed in saline group. **i** Schematic Structure of voltage-gated calcium channel located on cell membrane (left top). Representative immunofluorescence showing Cav 3.1 (left bottom), Cav 3.2 (right top), and Cav 3.3 (right bottom) expression in dmVMH of control and anxiety mice respectively, significantly increased Cav3.1 expression observed after chronic stress (Cav3.1^+^ cells counting, unpaired Student’s *t*-test, *P* < 0.001). Scale bar: 100 μm. **j** Quantification of T-VGCCs expression in dmVMH tissue between control (*n* = 5 mice) and anxiety groups (*n* = 6 mice). Expression of Cav 3.1 was significantly upregulated under chronic stress conditions (unpaired Student’s *t*-test, *P* = 0.0232). **k** Single-cell qRT-PCR analysis of T-VGCC expression in dmVMH neuronal subtypes between control (*n* = 16 cells) and anxiety groups (*n* = 13 cells). Expression of Cav 3.1 in burst firing neurons was significantly upregulated in anxiety group (unpaired Student’s *t*-test, Cav3.1, *P* = 0.0164). means ± SEM. *P < 0.05, **P < 0.01.

Bursting is an important firing pattern in neural systems and is essential for specific information transmission and function regulation [32]. The T-VGCC, including its three isoforms (Cav3.1, Cav3.2, and Cav3.3), is a pacemaker that can generate low-threshold spikes [28, 34]. Unlike hyperpolarization -activated cyclic nucleotide-gated channel (HCN), the T-VGCC can induce higher frequency firing and has a threshold near to RMP. We examined whether the burst firing in VMH relies on the function of T-VGCC by applying mibefradil, an antagonist of T-VGCC in whole-cell recording experiments. Results indicated that burst firing of dmVMH neurons elicited by a 10-pA current injection (silent and tonic-firing neurons failed to evoke burst firing with cosine current injection) was inhibited (Fig. 4a, Supplementary Fig. 6). Furthermore, we found that application of mibefradil increased the onset time in the dmVMH neurons of the anxiety group, but not in the control group, and the change in RMP was similar between the two groups (Supplementary Fig. 6). We also investigated the effects of mibefradil on the frequency-current curves using the same stimulus protocol described in Supplementary Fig. 2, and found that the firing rate of bursting neurons, especially in anxiety group, was decreased by application of mibefradil (Fig. 4b, right). Mibefradil exerted no obvious influence on the firing rate and subthreshold membrane potential of silent or tonic-firing neurons (Supplementary Fig. 6). These data support that the T-VGCC mediates burst firing in the dmVMH. We also found that blockade of NMDA receptors, but not AMPA receptors, significantly inhibited evoked-burst firing (Supplementary Fig. 7), which suggested a link between NMDA receptor and T-VGCC-mediated burst firing in VMH.

To investigate the in vivo effects of T-VGCC blockade, we bilaterally implanted a cannula and precisely delivered mibefradil or saline into the dmVMH of stressed mice to block burst firing (Fig. 4c). Results showed that mibefradil infusion increased the time spent in the central area of the open field and open arm of the EPM while no obvious difference observed in the control group treated with saline (Fig. 4d). We also investigated energy metabolic changes and food intake after administration of mibefradil, we found that administration of mibefradil significantly rescued the decreased RER, EE and food intake in stressed mice (Fig. 4e–h). Taken together, these data indicate that the T-VGCC in the dmVMH mediates chronic stress-induced behavioral and energy expenditure changes.

The expression of T-VGCC is highly correlated with T-type calcium currents, which directly affect the strength and width of bursting [31, 35]. To figure out which subtypes of T-VGCC contribute to the enhancement of burst firing after chronic stress, we applied immunostaining and qRT-PCR experiments to quantify the expression of three subtypes in VMH of control and stressed mice. Immunostaining showed that Cav3.1, Cav3.2, and Cav3.3 all expressed in the dmVMH of control and anxiety group, however the signals of Cav3.1 were much stronger in the dmVMH of anxiety group, compared with that of control group (Fig. 4i and Supplementary Fig. 8). To further confirm the differential expression of the T-VGCC subtypes after chronic stress, we performed qRT-PCR in dmVMH tissue to quantify T-VGCC expression. The expression of Cav3.1 in the dmVMH of stressed mice was significantly higher than that of the control group, whereas no obvious changes were observed in Cav 3.2 or Cav 3.3 (Fig. 4j). Moreover, we collected single neurons after whole-cell recordings for T-VGCC quantification, we combined the single-cell qRT-PCR and electrophysiological classification data to determine the differential expression of Cav3.1 in the dmVMH neuronal subtypes. Results showed that Cav3.1 expression was much more enriched in dmVMH bursting neurons after chronic stress (Fig. 4k). These data consistently demonstrated that T-VGCC subtype Cav3.1 mainly mediated the burst firing in dmVMH.

### Knockdown of Cav3.1 in dmVMH decreased burst firing and ameliorated chronic stress response

**Fig. 5:**
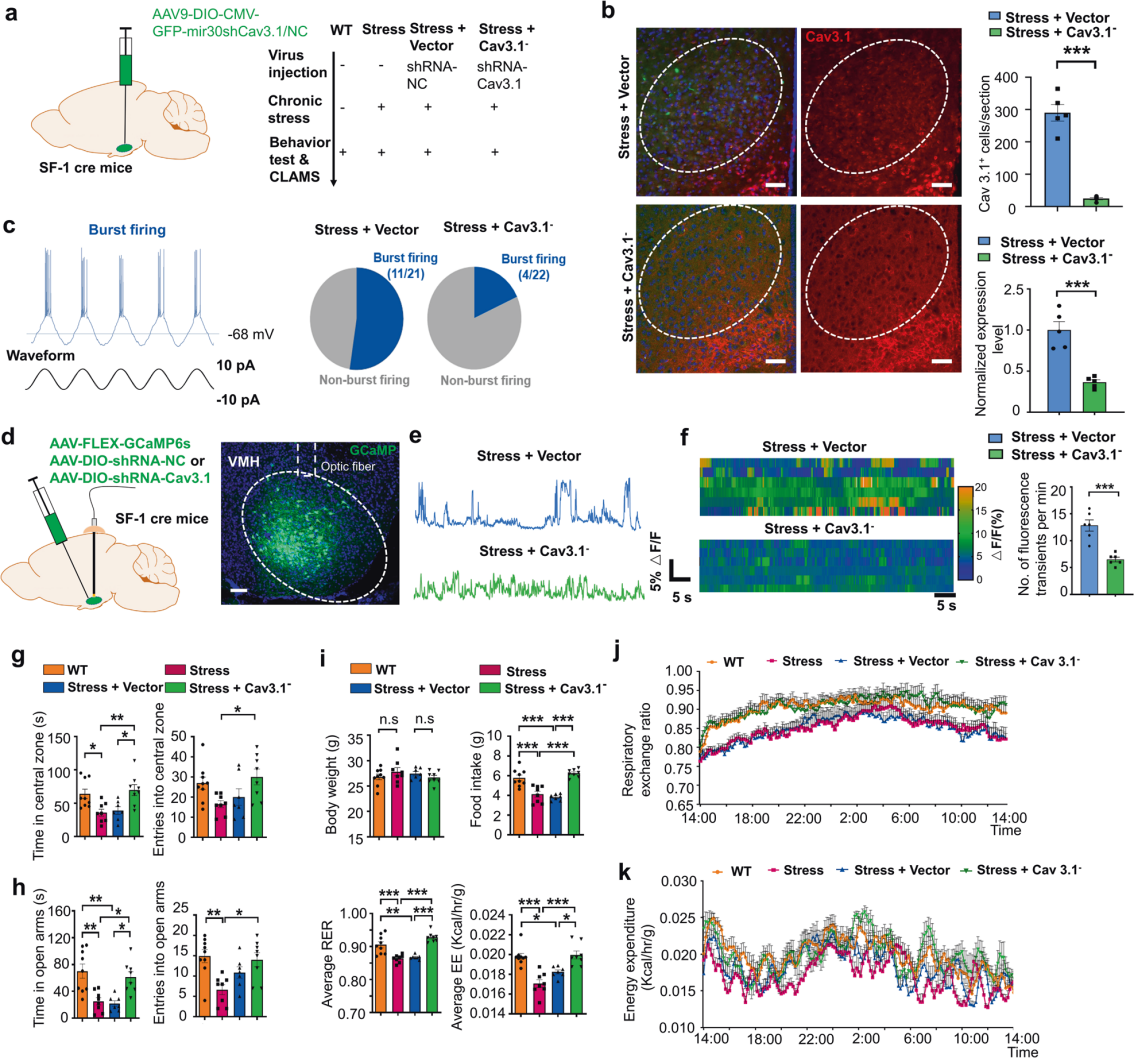
Knockdown of Cav3.1 in dmVMH decreased burst firing, and rescued anxiety-like behavior and metabolic alteration induced by chronic stress. **a** Schematic demonstrates injection of DIO-based shRNA-expressing AAV viral vector into dmVMH of SF-1 cre mice to interfere with Cav 3.1 expression. **b** Immunostaining of Cav 3.1 in stressed mice injected with shRNA-NC (negative control) or shRNA-Cav3.1 AAV vector. Scale bar is 100 μm. Cell counting (right, up) and gene expression analysis (right bottom) indicated an effective knock-down of Cav3.1 expression in stress + Cav3.1^−^ group (Cav3.1^+^ cells counting, unpaired Student’s *t*-test, *P* < 0.001; Q-PCR, unpaired Student’s *t*-test, *P* < 0.001). **c** Proportion of burst firing neurons decreased in stress + Cav3.1^−^ group (4/22) compared with stress + vector group (11/21). **d** Schematic of injection of GCaMP6s AAV viral vector into dmVMH of SF-1 cre mice to achieve in vivo photometry recording. Scale bar, 100 µm. **e** Representative traces of spontaneous calcium transients in VMH of stress + vector and stress + Cav3.1^−^ groups. **f** Heatmaps demonstrate different spontaneous GCaMP6s fluorescence signals in stress + vector and stress + Cav3.1^−^ groups (6 mice in each group, unpaired Student’s *t*-test, *P* < 0.001). **g** Time spent in central area and number of entries into central area of open field in WT, stress, stress + vector and stress + Cav3.1^−^ groups. Residence time: *P* = 0.0024; WT (*n* = 9) versus stress group (*n* = 8), *P* = 0.0324; stress + vector (*n* = 7) versus stress + Cav3.1^−^ group (*n* = 8), *P* = 0.0261; stress versus stress + Cav3.1^−^ group, *P* = 0.0084. Number of entries: *P* = 0.0024; WT versus stress group, *P* = 0.1795; stress + vector versus stress + Cav3.1^−^ group, *P* = 0. 2843; stress versus stress + Cav3.1^−^ group, *P* = 0.0433. (one way ANOVA, with Bonferroni correction). **h** Time spent in open arms and number of entries into open arms of elevated plus-maze in WT, stress, stress + vector and stress + Cav3.1^−^ groups. Residence time: *P* = 0.0002; WT (*n* = 9) versus stress group (*n* = 8), *P* = 0.0019; stress + vector (*n* = 7) versus stress + Cav3.1^−^ group (*n* = 8), *P* = 0.0142; WT versus stress + vector group: *P* = 0.0013; stress versus stress + Cav3.1^−^ group, *P* = 0.0219. Number of entries: *P* = 0.0025; WT versus stress group, *P* = 0.0031; stress + vector versus stress + Cav3.1^−^ group, *P* = 0.9999; stress versus stress + Cav3.1^−^ group, *P* = 0.0121 (one way ANOVA, with Bonferroni correction). **i** No significant differences in average body weights of WT, stress, stress + vector and stress + Cav3.1^−^ groups were observed after 4 weeks of chronic stress (*P* = 0.4755); WT (*n* = 9) versus stress group (*n* = 8), *P* = 0.9999; stress + vector (*n* = 7) versus stress + Cav3.1^−^ group (*n* = 8), *P* = 0.9999; Food intake: *P* < 0. 0001; WT versus stress group, *P* = 0.0001; stress + vector versus stress + Cav3.1^−^ group, *P* < 0.0001; WT versus stress + vector group, *P* < 0.0001; stress versus stress + Cav3.1− group, *P* < 0.0001. Average RER: *P* < 0.0001; WT versus stress group, *P* = 0.0003; stress + vector versus stress + Cav3.1^−^ group, *P* < 0.0001; WT versus stress + vector group, *P* = 0.0016; stress versus stress + Cav3.1− group, *P* < 0.0001. Average EE: *P* < 0.0001; WT versus stress group, *P* < 0.0001; stress + vector versus stress + Cav3.1^−^ group, *P* = 0.0112; WT versus stress + vector group, *P* = 0.0189; stress versus stress + Cav3.1− group, *P* < 0.0001 (one way ANOVA with Bonferroni correction). **j**, **k** 24-h RER and EE curve of WT, stress, stress + vector and stress + Cav3.1^−^ groups; For RER: two-way ANOVA: WT versus stress group, *P* = 0.0251, *F*(1, 15) = 6.186; stress + vector versus stress + Cav3.1^−^ group, *P* = 0.0289, *F*(1, 13) = 6.027; 8 mice in each group; For EE: two-way ANOVA: WT versus stress group, *P* < 0.001, *F*(1, 15) = 31.40; stress + vector versus stress + Cav3.1^−^ group, *P* = 0.0062, *F*(1, 13) = 10.654). All two-way ANOVA are performed with Bonferroni correction. Data are means ± SEM, *P < 0.05, **P < 0.01, ***P < 0.001.

Given the important role of Cav 3.1 in mediating burst firing in the dmVMH, we next tested whether down-regulation of Cav 3.1 expression in the dmVMH was sufficient to ameliorate anxiety-like behavior and energy metabolic changes induced by chronic stress. We developed a DIO-based RNAi method to specifically interfere with expression of Cav3.1 in SF-1 neurons under chronic stress, with the Cav3.1-shRNA or control vectors injected into the dmVMH bilaterally prior to chronic-stress exposure (Fig. 5a). Firstly, we used immunofluorescence to confirm the efficient knockdown of Cav3.1 in the dmVMH of stressed mice (Fig. 5b). The effects of Cav3.1 knockdown on burst firing were tested using whole-cell recording on brain slices obtained from Cav 3.1 knockdown mice. Results showed that 11 out of 21 dmVMH neurons in stress + vector group displayed evoked burst firing, whereas only 4 out of 22 dmVMH neurons displayed burst firing in stress + Cav3.1^−^ group (Fig. 5c), suggesting burst firing were inhibited by RNAi of Cav3.1. We also confirmed the effect of shRNA-Cav3.1 on the neural activity in VMH in vivo by applying calcium signal photometry recording (Fig. 5d). Spontaneous calcium activity of SF-1 neurons was suppressed in stress + Cav3.1^−^ group when compared with stress + vector^−^ group (Fig. 5e, f), suggesting that a down-regulation of Cav 3.1 expression was sufficient to inhibit the neural burst firing in the dmVMH.

To investigate phenotypic changes in these Cav3.1 knockdown mice, we carried out behavioral and energy metabolic tests after viral expression of shRNA of Cav3.1 for 4–5 weeks. Importantly, behavioral tests showed that Cav 3.1 knockdown significantly increased residence time and entries into the central area of the open field and open arm of the elevated plus-maze of stressed mice (Fig. 5g, h), suggesting that local knockdown of Cav 3.1 in the dmVMH was sufficient to rescue stress-induced anxiety-like behavior. For energy metabolic tests, we fasted all mice overnight and analyzed energy expenditure using CLAMS. Our data showed that Cav 3.1 knockdown effectively rescued the chronic stress-induced decrease in RER (Fig. 5i, j), as well as the reduction in food intake (Fig. 5i) and energy expenditure (Fig. 5k). No obvious body weight differences were observed among the control, stress, and RNAi groups (Fig. 5i).

Together with our optogenetic manipulation experiments, our results consistently demonstrated that Cav 3.1 in the dmVMH was both sufficient and necessary to elicit burst firing of dmVMH neurons. Furthermore, knockdown of Cav 3.1 in the dmVMH ameliorated chronic stress-induced phenotypic abnormalities, including anxiety-like behavior, lower RER, and decreased food intake.

### Anxiolytics inhibited Cav3.1 expression and burst firing in VMH to rescue energy expenditure changes

**Fig. 6:**
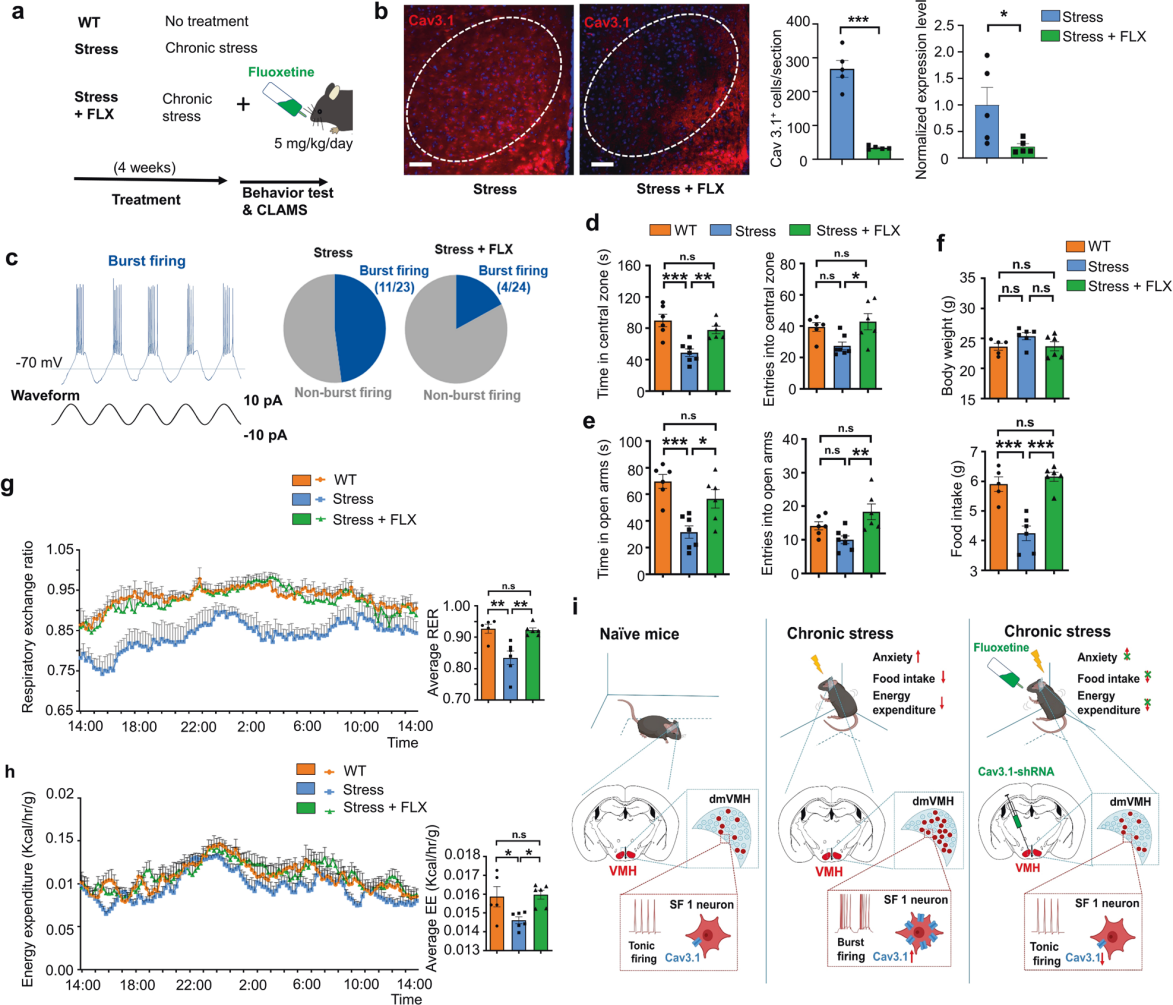
Anxiolytics suppressed Cav3.1 expression and burst firing in VMH, and rescued anxiety-like behavior and metabolic alteration induced by chronic stress. **a** Schematic demonstrates fluoxetine application and related experimental strategy. **b** Immunostaining of Cav 3.1 in mice undergo chronic stress alone or chronic stress plus fluoxetine treatment. Scale bar is 100 μm. Quantification of Cav3.1 expression (right) in these two groups (Cav3.1^+^ cells counting, unpaired Student’s *t*-test, *P* < 0.001; qRT-PCR, Mann–Whitney test, *P* = 0.0397). **c** Proportion of burst firing neurons decreased in stress + FLX (fluoxetine) group (4/24) compared with stress group (11/23). **d** Time spent in central area and number of entries into central area of open field in WT, stress, and stress + FLX groups. Residence time: *P* = 0.0004; WT (*n* = 6) versus stress group (*n* = 7), *P* = 0.0004; stress versus stress + FLX group (*n* = 6), *P* = 0.0091. Number of entries: *P* = 0.0160; WT versus stress group, *P* = 0.0827; stress versus stress + FLX group, *P* = 0.0209 (one way ANOVA with Bonferroni correction). **e** Time spent in open arms and number of entries into open arms of elevated plus-maze in WT, stress, and stress + FLX groups. Residence time: *P* = 0.0006; WT (*n* = 6) versus stress group (*n* = 7), *P* = 0.0005; stress versus stress + FLX group (*n* = 6), *P* = 0.0169. Number of entries: *P* = 0.0075; WT versus stress group, *P* = 0.2547; stress versus stress + FLX group, *P* = 0.0062 (one way ANOVA with Bonferroni correction). **f** No significant differences in average body weights of WT, stress, and stress + FLX groups were observed after 4 weeks of treatments, *P* = 0.1334; WT (*n* = 5) versus stress group (*n* = 6), *P* = 0.1890; stress versus stress + FLX group (*n* = 6), *P* = 0.1869. Food intake of mice in WT, stress, and stress + FLX group: *P* < 0.0001; WT versus stress group, *P* = 0.0003; stress versus stress + FLX group, *P* < 0.0001. (one way ANOVA with Bonferroni correction). **g**, **h** 24-h RER and EE curve of WT, stress and stress + FLX groups; For RER: two-way ANOVA: WT versus stress group, *P* = 0.0060, *F*(1, 9) = 12.8; stress versus stress + FLX group, *P* = 0.0030, *F*(1, 10) = 15.19; For EE: two-way ANOVA: WT versus stress group, *P* = 0.0375, *F*(1, 9) = 5.939; stress versus stress + FLX group, *P* = 0.0010, *F*(1, 10) = 20.89. Average RER of WT, stress, and stress + FLX group: one way ANOVA with Bonferroni correction, *P* = 0.0013; WT versus stress group, *P* = 0.0034; stress versus stress + FLX group, *P* = 0.0036. Average EE of WT, stress, and stress + FLX group: one way ANOVA with Bonferroni correction, *P* = 0.0169; WT versus stress group, *P* = 0.0463; stress + vector versus stress + FLX group, *P* = 0.0230. **i** Schematic showing that chronic stress enhanced burst firing and Cav3.1 expression in dmVMH neurons to induce the anxiety-like behavior and energy expenditure changes, which could be rescued by knocking down of Cav3.1 or application of anxiolytics. All two-way ANOVA are performed with Bonferroni correction. Data are means ± SEM, *P < 0.05, **P < 0.01, ***P < 0.001.

Fluoxetine is a serotonin selective reuptake inhibitor and has been widely used in the clinical treatment of anxiety disorders [36, 37]. Given the tight relation between Cav3.1 expression level and chronic stress-induced anxiety state, we hypothesize that anxiolytic drugs like fluoxetine might regulate the expression of Cav3.1 and burst firing in dmVMH. To test this hypothesis, we applied fluoxetine (5 mg/kg, oral) daily during the 4-weeks chronic stress, and then examined its effect on anxiety mice (Fig. 6a). Immunostaining demonstrated a decrease of Cav3.1 fluorescence signal after application of fluoxetine compared with stress group (Fig. 6b). qRT-PCR also suggested the expression of Cav3.1 decreased in the stress + FLX (fluoxetine) group (Fig. 6b), indicating that daily administration of fluoxetine effectively suppressed the Cav3.1 expression in VMH. Previous research has suggested that cellular signaling pathways, such as cAMP-protein kinase A (PKA), are involved in the regulation of Cav3.1 expression [38]. Thus, we tested the cAMP-PKA signals in stressed mice. Results indicated that cAMP, PKA, and CREB (cAMP-response element binding protein) were elevated in the dmVMH after chronic stress, but application of FLX blocked this effect, thus suggesting the possible mechanism underlying the regulation of Cav3.1 expression by FLX (Supplementary Fig. 9b). We also applied in vitro patch clamp recording to test whether the burst firing of VMH neurons is impaired by the fluoxetine. Result showed that 11 out of 23 dmVMH neurons from stressed mice displayed evoked burst firing, whereas only 4 out of 24 dmVMH neurons from stress + FLX group succeeded (Fig. 6c), suggesting that burst firing in dmVMH was inhibited by daily application of fluoxetine. Moreover, application of FLX by aCSF infusion also inhibited the spiking frequency of single burst of stressed mice (Supplementary Fig. 9a).

To further confirm the anxiolytics effect on our chronic stress mice model, we also performed behavioral tests and CLMAS experiment. Results of behavioral tests showed that application of fluoxetine in stressed mice significantly increased the residence time and entries into the central area of the open field and open arm of the elevated plus-maze (Fig. 6d, e). Data from CLAMS also showed that fluoxetine effectively rescued the chronic stress-induced decrease in RER (Fig. 6f, g), as well as the reduction in food intake (Fig. 6f) and energy expenditure (Fig. 6f, h). No obviously effect was observed in the body weight, which is consistent with our result of RNAi experiment (Fig. 6f). Taken together, daily administration of fluoxetine can effectively rescue the anxiety-like behavior and metabolic alteration caused by chronic stress, our data supported that the suppression of Cav3.1 expression and burst firing in VMH may be a potential mechanism underlying its anxiolytics effects.

## Discussion

During the evolution of the animals, persistent exposure to stressors exert pressure on central nervous system to integrate the regulation of energy homeostasis and the behavior response to external environment [1, 3, 4]. Previous studies have indicated that the dmVMH is an important stress coping center to balance aversive behavior and energy seeking [2], and may be a possible hub connecting stress-induced emotion and energy expenditure. Here, we reported that chronic stress-induced burst firing enhancement in the dmVMH, which was critical for inducing anxiety-like behavior and energy expenditure alteration in stressed mice. Our findings demonstrated that dmVMH burst firing neurons play an important role in connecting the emotional state of anxiety and energy homeostasis, Cav 3.1 is essential for burst firing in dmVMH, and knockdown of Cav3.1 or administration of fluoxetine rescued anxiety-related energy expenditure changes through inhibition of burst firing in dmVMH (Fig. 6i).

The innate defense behavior in the aversive environment and simultaneous adjustment of energy expenditure is essential for stress coping and survival in animals, whereas the long term chronic unpredictable stress is detrimental to both mental and physical health. Up to now, how these physiological processes integrate in the brain and the underlying neural mechanisms remain poorly understood. The dmVMH is implicated in integrating information in the limbic system and in maintaining energy balance [39]. Furthermore, SF-1 neurons in the dmVMH constitute a nutritionally sensitive switch, which modulates the competing motivations of feeding and avoidance of environments full of acute stress [2]. Given the important role of the dmVMH in balancing acute stress inputs and food-seeking, the effects of persistent unpredictable stress on the function of dmVMH neurons require further research.

In our study, the dmVMH was confirmed to be involved in integrating chronic stress inputs to regulate anxiety and energy expenditure. We established an unpredictable and persistent stress mouse model and characterized the model with multiple energy metabolic, electrophysiological, and behavioral approaches. Consistent with previous reports [11], we found that unpredictable persistent stress-induced aversive behavior, decreased food intake, and shifted the RER toward fat oxidation. Our in vivo electrophysiology recording showed enhanced neural activity in theta band after chronic stress. In vivo photometry further demonstrated that the spontaneous calcium signal in SF-1 neurons was stronger and lasting longer in the stress group than in the control group. SF-1 neurons have been implicated in connecting with other neurons in VMH and form a complex network to cope with specific aversive stimuli [26]. Our results support this and suggested that the aversive stimulus-processing network may have been changed after long term repeated stress.

To dissect the alteration in dmVMH neuronal activity under chronic stress conditions, we studied the electrophysiology of dmVMH neurons and classified the dmVMH neurons into three subtypes: i.e., silent, tonic-firing, and bursting. By comparing these subtypes in the control and stress groups, we found an enhancement of burst firing in the dmVMH after chronic stress, including increased percentage of burst firing neurons, spikes of single burst, and decreased ISI of bursts. Our data demonstrated that chronic stressors induced obvious electrophysiological changes in dmVMH neurons at the cellular level, further supporting the important role of the dmVMH in chronic stress.

Many previous studies have indicated that burst firing of specific neuronal subpopulations is critical for performing specific functions. In the hippocampus, a single burst can produce long-term synaptic modifications [40]. In the lateral habenula, bursting activity depends on the NMDA receptor and T-VGCC; and most importantly, can drive behavioral aversion and depression-like symptoms [28]. We found that 32.94% of neurons were able to fire in a burst pattern (mediated by Cav3.1) in the dmVMH, whereas the other subtypes lacked the ability to fire bursts, even with cosine current injection. Neuronal burst firing neurons in the dmVMH is actively involved in chronic stress response, and the ISI of burst firing was significantly correlated with the anxiety-like behavior in anxious mice after chronic stress.

To mimic burst firing in vivo, we applied optogenetic inhibition with an NpHR-expressing AAV vector to elicit burst firing in yellow light illumination intervals [28, 41], and we found that burst firing in the dmVMH could not be elicited under an illumination frequency greater than 0.2 Hz, indicating regional specificity of T-VGCC activation kinetics. In vivo photometry study showed that optogenetically induced burst firing can evoke long-lasting calcium activity in dmVMH. Based on the results of in vivo electrophysiology recording and photometry, we hypothesize that neural burst firing may facilitate the synchronous activity in VMH, and modulate the excitability of neuronal network in VMH during stress coping.

Importantly, we applied wireless optogenetics to successfully achieve burst manipulation in non-stressed mice residing within a closed metabolic cage, followed by energy metabolism analyses and behavioral open field and EPM tests. Our data indicated that optogenetic-elicited burst firing was sufficient to promote the chronic stress-induced phenotypes described above. Taken together, these data suggest that burst firing can be evoked by optogenetics in the dmVMH, and this change in neuronal firing pattern is critical for integrating anxiety-like emotional state and energy expenditure regulation.

T-VGCC is widely expressed in the central nervous system and can be activated by stimuli near the RMP to elicit burst firing. Three isoforms of T-VGCC genes: i.e., Cav3.1, Cav3.2, and Cav3.3, make distinct contributions to cellular electrophysiological properties [34, 35, 42]. In our study, we identified the existence of Cav3.1, Cav3.2, and Cav3.3 in the dmVMH region. T-VGCC antagonist mibefradil blocked burst firing of dmVMH neurons, thus suggesting T-VGCC-dependent burst firing in the dmVMH. Based on the combined analysis of electrophysiological classification and single-cell qRT-PCR, we found that Cav3.1 expression increased under chronic stress, which contributed to the enhancement of burst firing in the dmVMH.

We further investigated the necessity of Cav3.1 in chronic stress-induced anxiety-like behavior and energy metabolic disorders. We found that microinjection of mibefradil ameliorated anxiety-like behavior. Using RNAi methods, the expression of Cav3.1 in the dmVMH was specifically knocked down, resulting in the significant inhibition of neuronal bursting activity. Behavioral and energy expenditure experiments further demonstrated that the chronic stress responses described above were partially rescued through RNAi. Thus, our findings consistently demonstrated that Cav3.1 may be a potential drug target for the treatment of anxiety and related energy metabolic disorders.

Importantly, we also found that long-term application of fluoxetine inhibited VMH Cav3.1 expression and suppressed neural burst firing under chronic stress. Fluoxetine is an anxiolytics drug and also inhibitor of serotonin reuptake [36], our new findings of its effects on Cav3.1 expression and burst firing put forward a possible mechanism explaining its anxiolytic effect, and also broaden its use in the intervention of metabolic disorders. Moreover, we found that fluoxetine decreased the spiking rate per burst, which is consistent with previous studies that fluoxetine could suppressed the calcium influx through Cav3.1 [43].

The current study has several limitations. Firstly, we focused on exploring neuronal activity changes in the dmVMH after chronic stress, further research should focus on identifying the upstream mechanism regulating Cav3.1 expression, especially the receptors in dmVMH regulating Cav3.1 expression. Several factors might contribute to the increased percentage of burst firing neurons after stress, including stress-induced hormones or feeding-related peptides [25]. Previous studies have reported that estrogen can regulate the expression and function of T-VGCC in ventrolateral VMH neurons through estrogen receptors [44], and noradrenaline receptor-PKA signals can regulate Cav3.1 expression in pinealocytes [38]. Our study indicated that fluoxetine inhibited the elevation in Cav3.1 expression after chronic stress. This effect could act via cAMP-PKA signaling suppression, or inhibition of calcium influx through Cav3.1. However, our data did not exclude the possibility that other signaling pathways may also be involved in the regulation of Cav3.1 expression.

Secondly, GABAergic neural circuits and astrocytes have been found in the VMH and are implicated in regulating anxiety and metabolism, and the frequency of inhibitory postsynaptic current (IPSC) of VMH neurons increase after chronic stress [45–47]. Optogenetically induced inhibitory inputs caused burst firing in thalamocortical neurons [48], thus enhanced IPSC input may promote the generation of burst firing in dmVMH neurons after chronic stress. We also demonstrated that glutamate receptors (especially NMDA receptors) affect the generation of burst firing, as blockade of NMDA receptors significantly inhibited burst firing evoked by current injection and caused membrane hyperpolarization. However, whether synaptic glutamate uptake mediated by astrocytes affects burst firing requires further study. Moreover, as the function of the T-VGCC is highly membrane potential-dependent, how upstream inputs integrate with the surrounding microenvironment to regulate burst firing needs to be further explored.

In summary, our study identified bursting firing neurons in the dmVMH as a crucial hub regulating emotional state and energy metabolic disorders. We identified Cav 3.1 as the crucial regulator of bursting firing of dmVMH neurons, and this neuronal firing pattern changes might explain the simultaneous alterations of innate behavior and energy expenditure during the stress coping of animals. Our findings provides a more complete understanding of the chronic stress-induced emotional malfunction and peripheral metabolism disorders, and also reveal a potential therapeutic target for treating such malfunctions.

## Supplementary information

Supplementary Material and Methods

Supplementary tables and figures
