# Supplementary material for: Cav3.1-driven bursting firing in ventromedial hypothalamic neurons exerts dual control of anxiety-like behavior and energy expenditure
Source: Mol Psychiatry. 2022 Mar 22;27(6):2901–13. doi: 10.1038/s41380-022-01513-x (PMC9156408; doi:10.1038/s41380-022-01513-x)
Supplement: Supplementary file 1 — Supplementary Material and Methods [file 41380_2022_1513_MOESM1_ESM.pdf]

Cav3.1-driven bursting firing in ventromedial hypothalamic neurons exerts dual control of anxiety-like behavior and energy expenditure

Jie Shao[1, 2]†, Da-Shuang Gao[1, 2]†, Yun-Hui Liu[1]†, Shan-Ping Chen[1], Nian Liu[1], Lu Zhang[1], Xin-Yi Zhou[1], Qian Xiao[1], Li-Ping Wang[1], Hai-Lan Hu[3], Fan Yang[1,2]*

Supplementary Materials and Methods

Animals

All procedures were carried out in accordance with protocols approved by the Ethics Committee of the Shenzhen Institutes of Advanced Technology, Chinese Academy of Sciences. Male C57BL/6 mice (4-weeks old) were purchased from the Guangdong Medical Laboratory Animal Center (Guangzhou, China). The SF-1-Cre mice (stock no: 012462, male) were obtained from Jackson Laboratories (Bar Harbor, ME, USA). Mice were housed at 22–25 °C on a circadian cycle of 12-hour light and 12-hour dark with ad-libitum access to food and water.

Chronic stress procedures

All animals (4-weeks old, unless stated elsewhere) used in this study were male, and randomly assigned to either control or stress groups by someone who blinded to following experiments for chronic stress study. The stress group was daily subjected to one stressor which randomly chosen from following: (i) 10 mice squeezing in a relatively small cage (15 cm×10 cm×4 cm) for two hours. (ii) wet bedding in home cage overnight (200 ml water was added to moisten bedding). (iii) each mouse was tightly restraint in a tube for two hours. The chronic stress protocol lasted for 28

consecutive days (unless stated elsewhere). Mice in different stress groups received the same number of each stressor. Control animals were subjected to no stressors. After chronic stress, all mice were performed behavior test the next day by investigator who blinded to the group allocation. Mice were sacrificed for *c-fos* staining at 1h after behavior tests finished.

Behavioral tests

Elevated plus maze (EPM). Exposure (5-min) to EPM was used to assess locomotor activity and anxiety-related behaviors after chronic stress and other treatment, and experimenter was blind to these treatments. The stress and control groups (n = 10 - 11/group) were placed in the center of the plus maze facing an open arm and behavior was recorded for the entire 5 min using an overhead-mounted camera. Videos recorded during the EPM test were analyzed with ANY-maze software (Stoelting Co., Wood Dale, USA) to acquire data on time spent in open and closed arms, locomotor activity (total distance travelled in maze), and entries into the open arm. Anxiety-related behavior is associated with less exploration in the open arm relative to overall exploration of all arms.

Open field test (OFT). Open field exposure (10-min) was performed to assess locomotor activity and anxiety-related behaviors by experimenter was blind to previous treatments. Mice (n = 10 - 11/group) were placed in the center of an open field and behavior was recorded for the entire 10 min. Videos were analyzed to acquire data on time spent in

the center and corner areas, total locomotor activity, and number of entries into the center area. Anxiety-related behavior is associated with less exploration of the center area.

CLAMS and energy expenditure

To characterize metabolic changes caused by chronic stress or other treatments, RER was measured by indirect calorimetry using a four-chamber open-circuit calorimeter (Oxymax Series; Columbus Instruments, Columbus, OH, USA). Mice were food deprived overnight, with body weight and chow in each cage weighed prior to the experiment. During the experiment, mice were housed individually in specially built Plexiglas cages (40 × 25 × 20 cm). Temperature was maintained at 22 °C with an airflow of 0.5 per min. Food and water were available *ad libitum*. Mice were subsequently monitored in the system for 24 h (whole light-dark cycle). Oxygen consumption (VO2) and carbon dioxide production (VCO2) were measured every 10 min. The RER was calculated as a quotient of VCO2/VO2, with 1 representing 100% carbohydrate oxidation and 0.7 representing 100% fat oxidation. Energy expenditure (kcal heat produced) was calculated as calorific value (CV) × VO2, where CV is 3.815 + 1.232 × RER [1]. Metabolic data collected from the 24-h monitoring period were averaged for energy expenditure and RER. After the experiment, chow in each cage was weighed to calculate food intake.

Slice preparation

Mice were deeply anesthetized with isoflurane and decapitated rapidly. Brains were then removed and transferred to chilled cutting solution within 3 min. Cutting solution contained (in mM): choline chloride 110; KCl 2.5; Na-pyruvate 0.6; $MgCl_2$ 7.0; $CaCl_2$ 0.5; NaH_2PO_4 1.3; $NaHCO_3$ 25; glucose 20 (pH 7.4). The chilled cutting solution was bubbled with carbogen (95% O_2 and 5% CO_2) for at least 30 min before use. Coronal slices (250–300 mm thick) were prepared on a vibratome (Series 1000, Warner Instruments, Berlin, Germany), and incubated in artificial cerebrospinal fluid (ACSF) containing (in mM): NaCl 125; KCl 2.5; Na-pyruvate 0.6; $MgCl_2$ 1.3; $CaCl_2$ 2.0; NaH_2PO_4 1.3; $NaHCO_3$ 25; glucose 10 (pH 7.4) bubbled with carbogen at 34 °C for 30 min. After incubation, all slices were equilibrated in ACSF at room temperature (24–26 °C) for at least 40 min. Single slices were placed on the recording chamber perfused with ACSF bubbled with carbogen at room temperature. Unless stated otherwise, drugs were applied with perfused ACSF.

Electrophysiology

Whole-cell patch clamp recording was performed on VMH neurons by experimenter blind to the treatment of mice. The dmVMH was identified based on landmarks (third ventricle). Recordings were obtained with multi-clamp 700B amplifiers (Molecular Devices, San Jose, USA) under visual guidance using a Nikon FN1 microscope (Tokyo, Japan). Electrophysiological data were acquired and analyzed using pClamp 10 software (Molecular Devices, San Jose, USA). Whole-cell recordings were performed

with borosilicate glass electrodes (0.69 mm OD, 5–7 MΩ) with internal solution containing (in mM): K-gluconate 135.0; KCl 4.0; NaCl 2.0; HEPES 10; EGTA 4.0; Mg-ATP 4.0; Na-GTP 5.0. Osmolality was adjusted to 290–310 mOsm kg^{-1} with sucrose and pH was adjusted to 7.4 with KOH.

After forming a high-resistance seal (GΩ), the cell was held in current-clamp mode for 7–10 min until access resistance stabilized. Resting membrane potential (RMP) was assessed at the beginning of the recording period after stabilization of access resistance, and periodically monitored throughout the recording by momentarily relieving the direct current injection. To elucidate differences among neurons, 800-ms current injections (-100 to 100 pA in 10 pA increments; 5 s interstimulus interval) were applied, and the number of action potentials evoked by each current injection, input resistance, and half-width of action potentials were determined.

Immunohistochemistry

Mice were first anesthetized by chloral hydrate (10% in saline, 1 ml/100 g, intraperitoneally), then perfused with 0.01 M PBS and 50 mL of 4% PFA transcardially. Brains were dissected and post-fixed in 4% PFA overnight and then transferred to 30% w/v sucrose solution for cryoprotection until sinking. Sections from the entire anterior-posterior range of the VMH were stained using an antibody specific to Cav3.1 (1: 100, Alomone labs, Cat# ACC-021, RRID: AB_2039779), Cav3.2 (1: 100, Alomone labs, Cat#ACC-025, RRID: AB_2039781), Cav 3.3(1: 100, Alomone labs, Cat# ACC-009,

RRID: AB_2039783), or *c-fos* (1: 200, Cell signaling, Cat# 4384, RRID:AB_2106617). Briefly, sections were washed, permeabilized in 0.1% Triton X-100/PBS for 15 min/three times, washed again, and blocked in 10% normal goat serum (NGS) (w/v)/0.1% Triton X-100/PBS for 30 min. Primary antibody was added, and sections were incubated overnight at 4 ℃. The following day, sections were washed with 0.01 mM PBS, incubated with secondary antibody (Alexa Fluor 594 Goat Anti-Rabbit, Cat#115-585-003, RRID: AB_2338059 or Alexa Fluor 488 Goat Anti-Rabbit, Cat#111-547-003 RRID: AB_2338058, Jackson) in 1% NGS/0.1% Triton X-100/PBS for 1 h at room temperature, then washed, mounted, and cover slipped with mounting medium containing DAPI (Vector labs, Burlingame, USA).

Drugs and reagents

Fluoxetine and all chemicals included in ACSF prescription were purchased from Sigma-Aldrich (Merck KGaA, Darmstadt, Germany). Mibefradil (10 μM) were purchased from Tocris (Bio-Techne, Minneapolis, USA) and used for electrophysiology and microinjection. NBQX (1,2,3,4-tetrahydro-6-nitro-2,3-dioxo-benzo[f]quinoxaline -7-sulfonamide, 30 μM), 5-HT (40 μM) and AP5 (2-amino-5-phosphono -pentanoic acid, 30 μM) were acquired from Med Chem Express (MCE, Shanghai, China).

ELISA tests for signaling pathway

After all treatment, mice of each group were anesthetized with isoflurane and brains were acute cut in cold phosphate-buffered saline (PBS) solution. Protein concentration

was determined using BCA kit (23225, ThermoFisher, US) after lysis by commercial cell lysis solution (P0013B, Beyotime Biotechnology, China) according to the manufacturer instructions. ELISA tests were performed using the following ELISA kits: Mouse cAMP ELISA Kit (KS13362, Shanghai KESHUN, China), Mouse PKA ELISA Kit (MM-44733M1, Shanghai MLBIO, China) and Mouse cAMP response element binding protein (CREB) ELISA Kit (CSB-E15860m, CUSABIO, China). All experiments are performed according manufacturer instructions.

Cell harvesting and single-cell Quantitative real-time polymerase chain reaction (qRT-PCR)

Single Cell-to-CT™ Kits (Thermo Fisher, USA) were used to perform single-cell qRT-PCR. After recording, single cells were collected after patch clamp recording and lysed to acquire total RNA. Mastercycler 5333 PCR thermal cycler (Eppendorf, Germany) was used to perform reverse transcription and pre-amplification. TaqMan gene expression assay was used to assay each gene. Gene expression levels were normalized to the expression of housekeeping gene *Rn18s*. All protocols were performed according to the manufacturer's instructions. qRT-PCR was performed with a Light cycler 480 (Roche, Switzerland).

Tissue collection and qRT-PCR

To acquire dmVMH tissue, mice were first anesthetized with isoflurane. After bathing in cold 1% diethyl pyrocarbonate (DEPC) phosphate-buffered saline (PBS) solution,

brains were acute cut on a vibratome. VMH tissues were dissected microscopically from those sections, and quickly transferred to TRIzol reagent. The manufacturer's standard protocols for RNA extraction (TransGene, China) and synthesis (TOYOBO, Japan) were followed. Primers used for qRT-PCR included: Cav 3.1 (5'-TGG CCTTCTTCGTCCTGAAC-3' and 5'-TTCTCCAGCCTCTTTAGTCGC-3'). Cav 3.2 (5'-CGGCCCTACT ACGCAGACTA-3' and 5'-TTAAGGGCCTCGTCCAGAGA-3'), Cav 3.3 (5'-CTGCTATTCTCCAGCCCAGG-3' and 5'-AGCTGCACCTCTTG CTTGT-3'). Expression of these gene was normalized to the expression of housekeeping gene β-Actin (*Act-b*).

Fiber photometry recording

Fiber photometry (ThinkerTech, Nanjing, China) [2] was utilized to record calcium activity in VMH. We injected 200 nL of AAV-Flex-GCaMP6s (Taitool Bioscience, Shanghai, China) and implanted optical fiber (200 μm OD, 0.37 NA, Newdoom, Hangzhou, China) into the VMH of SF-1 cre mice. To figure out the difference in spontaneous calcium activity between control and stressed mice, GCaMP6s was injected into VMH after 4-weeks chronic stress and photometry recording has been preformed 4-weeks after surgery. Photometry recording and behavioral tests started at same time to synchronize the timeline of calcium signals and behavior video. Data recorded before and after mice entering anxiogentic contexts (central zone of open field and open arms of EPM) was analyzed. To test the effect of periodic optogenetic inhibition on the neural activity of SF-1 neurons, GCaMP6s was injected with AAV9-

DIO-NpHR-mCherry (AAV9-DIO-mCherry as control group, Brain VTA, Wuhan, China) into dmVMH. In the RNAi knockdown study, GCaMP6s was injected with shRNA-Cav3.1 vector (shRNA vector as negative control, WZ Bioscience, Jinan, China) into VMH of SF-1 cre mice prior to 4-weeks chronic stress. Mice were housed at least 3 weeks before experiments to ensure virus expression. The 473 nm and 580 nm laser beam were reflected off a dichroic mirror and focused by objective lens to excite GCaMP6s fluorescence and NpHR channels respectively. Calcium fluorescence signals was collected by a photomultiplier tube and then converted into electrical signals (100Hz). The $\Delta F/F$ values were calculated by $(F/Fmean - 1)\times 100\%$, Fmean was determined by average fluorescence intensity throughout recording. Calcium signal wave higher than $\mu + 3\sigma$ was regarded as a fluorescence transient (μ and σ represents the average and the standard deviation of the baseline signal) [3]. Recording traces were visualized using Prism 7 (GraphPad Software, La Jolla, CA, USA) and custom MATLAB (MathWorks, Natick, USA) scripts produced by Thinker Biotech.

In vivo electrophysiology

After 4-weeks chronic stress, mice were implanted with two nickel-chromium wires (25-μm diameter; AM Systems, Sequim, USA) targeting the dmVMH, connector was bind to wires and fixed on skull with dental cement. After recovery (1 week) from surgery, mice were returned to the recording sessions. Neuronal activity was collected using a Plexon Multichannel Acquisition Processor system (Plexon, Dallas, USA). Local field potentials (digitized at 1 kHz sampling rate, low-pass filtered up to 250 Hz)

were recorded simultaneously for 90 min with a gain of 5 000×. After the recording sessions, mice were anesthetized by pentobarbital sodium and perfused intracardially. The electrode recording position was marked by histological staining.

Stereotaxic surgery and viral injection

For all stereotaxic surgery, mice were anesthetized by pentobarbital sodium (0.3% in saline, 1 ml/100 g, intraperitoneally) and placed in a stereotaxic apparatus for surgery. Stereotaxic surgical procedures were performed using standard protocols. To target the dmVMH, bilateral brain injection coordinates relative to bregma were chosen (AP, -1.58 mm; ML, ±0.3 mm; DV, -5.5 mm). Unless stated otherwise, 0.2 μL of viral vector was injected into the VMH at a rate of 0.1 μL/min using a 10-μL Hamilton syringe and a syringe infusion pump (World Precision Instruments, USA).

For RNAi knockdown study, 5-weeks old SF-1 cre mice were divided into four groups, including control group (no treatment), stress group (chronic stress), stress + vector (chronic stress and AAV vector injected) and stress + Cav 3.1^{-} group (chronic stress and AAV-shRNA injected). Cav3.1-shRNA (AAV-CMV-GFP-shCACNA1Gmir30-bGHpA, WZ Bioscience, Jinan, China) or control vector (AAV-CMV-GFP-Scramble-bGHpA, WZ Bioscience, Jinan, China) was used to record calcium signals in VMH was injected into the VMH of mice in RNAi group before chronic stress. Mice were housed for five weeks (4-days recovery and 4-weeks chronic stress) before anxiety-like behavior and metabolic tests. To assess the knockdown effectiveness of Cav 3.1 shRNA, mice were

perfused with 4% paraformaldehyde (PFA) and brain tissues were removed for immunostaining analysis after the final session.

For microinjection, a stainless-steel guide-cannula (0.6 mm outer diameter and 0.4 mm inner diameter, RWD, Shenzhen, China) was implanted into the diencephalon to target the VMH (AP, -1.58 mm; ML, ±0.3 mm; DV, -5.0 mm) after 4-weeks chronic stress. The guide-cannula was fixed to the skull using dental cement and three stainless steel screws. Each guide-cannula was sealed with a stainless-steel wire for protection from obstruction. Before behavioral testing, the stainless-steel wire was replaced with an injection cannula (0.5 mm deeper than guide-cannula to break astrocyte aggregation), through which the drug dissolved in saline (200 nl) was delivered at 100 nl/min into the VMH. After microinjection, mice were rested for 30 min before tests.

Wireless optogenetic manipulation

To achieve wireless optogenetic manipulation of burst firing neurons, AAV9-DIO - NpHR-mCherry or AAV9-DIO-mCherry (Brain VTA, Wuhan, China) was injected unilaterally into the VMH in the left hemisphere. Mice were housed for four weeks following injection for viral expression before initiation of experiments. A copper coil with a light emitting diode (LED) on the right side of the tip (Inper, Hangzhou, China) was implanted into the brain of NpHR-expressing mice, with the LED located 0.2 μm left of the dmVMH. The coil and indicator were fixed on the surface of the skull bone with Vetbond Tissue Adhesive (3M, USA). Charging the coil was achieved through

antennas surrounding the cage, and the antennas was adjusted to ensure ~10 mw/mm^2 power density of yellow light. To initiate burst firing of dmVMH neurons, 550-nm yellow light stimulation was performed at 0.1 Hz (2 s/pulse) in the NpHR-mCherry group during the metabolic (one illumination trial/12 h, each trial lasted for 2 h) and behavioral tests (OFT, 10 min; EPM, 5 min).

Statistical analysis

Initial sample sizes were established at n = 6/group at least which similar to our previous publication, and final sample size was adjusted to account for experiment interrupted or failure of acquiring complete data, such as virus or fiber implant missing. Slice electrophysiological data were analyzed with pCLAMP (Molecular Device, San Jose, CA, USA). *In vivo* electrophysiology data were analyzed with NeuroExplorer 5.0 (Plexon, Dallas, USA). All data were imported into Prism 7 (GraphPad Software, La Jolla, CA, USA).To verify the appropriateness of the following statistical analyses, normality was assessed using D'Agostino-Pearson test and Kolmogorov-Simimov test, and variance was compared using F test. Unless stated otherwise, the data are presented as means ± SEM. Statistical significance was determined using two-tailed unpaired Student's *t*-tests, Mann-Whitney U test (data did not follow normal distribution or not pass the F test) and one-way analysis of variance (ANOVA) with Bonferroni when comparing two groups or more, two-tailed paired Student's *t*-test when comparing the effects of different treatments in the same group. When multiple measures were compared between groups (e.g., current-frequency curves), repeated measures two-way

ANOVA with Bonferroni correction was used. Differences were considered significant at $P < 0.05$.

To classify dmVMH neurons, unsupervised cluster analysis was performed with SPSS v19 (Chicago, IL, USA) using squared Euclidean distances. The parameters for cluster analysis were chosen based on their lack of linear correlation with each other. The following electrophysiological parameters were included for analysis: onset time, evoked firing rate, input resistance, and potential sag by H-current (Hyperpolarization-activated current). Resting potential were excluded from the parameters for cluster analysis because of their linear correlation with onset times. All electrophysiological parameters were converted into standardized z-scores before clustering.

References

1. Hardaway JA, Halladay LR, Mazzone CM, Pati D, Bloodgood DW, Kim M *et al.* Central Amygdala Prepronociceptin-Expressing Neurons Mediate Palatable Food Consumption and Reward. *Neuron* 2019; **102**(5)**:** 1088.

2. Cao P, Chen C, Liu A, Shan Q, Zhu X, Jia C *et al.* Early-life inflammation promotes depressive symptoms in adolescence via microglial engulfment of dendritic spines. *Neuron* 2021; **109**(16)**:** 2573-2589.e2579.

3. Xia G, Han Y, Meng F, He Y, Srisai D, Farias M *et al.* Reciprocal control of obesity and anxiety-depressive disorder via a GABA and serotonin neural circuit. *Molecular psychiatry* 2021.
