# Supplementary material for: Cav3.1-driven bursting firing in ventromedial hypothalamic neurons exerts dual control of anxiety-like behavior and energy expenditure
Source: Mol Psychiatry. 2022 Mar 22;27(6):2901–13. doi: 10.1038/s41380-022-01513-x (PMC9156408; doi:10.1038/s41380-022-01513-x)
Supplement: Supplementary file 2 — Supplementary tables and figures [file 41380_2022_1513_MOESM2_ESM.pdf]

Cav3.1-driven bursting firing in ventromedial hypothalamic neurons exerts dual control of anxiety-like behavior and energy expenditure

Supplementary Information

This file includes:

Supplementary Fig. 1 to 9
Supplementary Table 1

Supplementary Table 1. Electrophysiological properties of three dmVMH neuronal subtypes

	Silent (n = 19)	Tonic-firing (n = 38)	Bursting (n = 28)
Input resistance (MΩ)	535.30 ± 21.83	661.72 ± 19.84	662.72 ± 24.21
Onset Time (ms, 50 pA)	37.17 ± 2.53	18.20 ± 1.28	25.84 ± 1.87
RMP (mV)	-65.27 ± 1.08	-57.50 ± 0.92	-63.39 ± 1.07
Overshoot by H-current (mv, -100 pA)	7.26 ± 1.17	10.71 ± 0.94	12.01 ± 1.19
Evoke firing rate (Hz, 100 pA)	17.53 ± 0.60	21.76 ± 0.50	22.00 ± 0.73
AHP (mV)	-12.38 ± 0.53	-15.2 ± 0.50	-14.26 ± 0.65
Half width (ms)	2.17 ± 0.08	2.00 ± 0.04	2.04 ± 0.05
AP amplitude (mV)	71.79 ± 1.88	73.29 ± 1.25	73.29 ±1.28

Data are represented as mean ± SEM. RMP: resting membrane potential; H-current: hyperpolarization-activated current; AHP: after-hyperpolarization potential; AP: action potential.

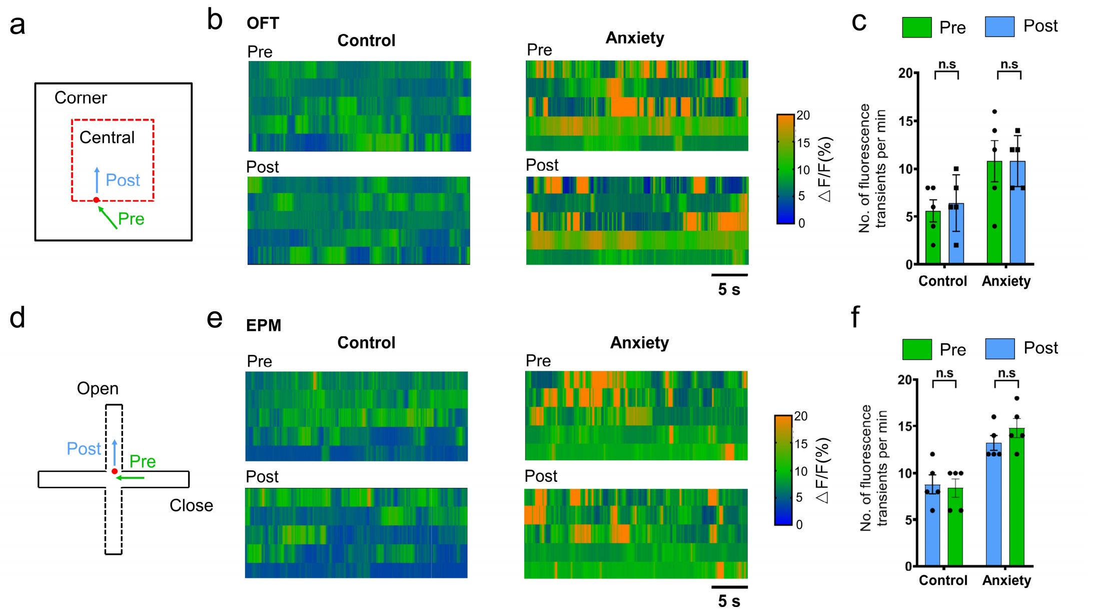

Supplementary Figure 1. *In vivo* photometry recording of dmVMH neurons in control and stressed mice during behavioral tests.

(**a**) Schematic of photometry recording during open field test (OFT). (**b**) Heat maps and (**c**) statistics analysis demonstrated no obvious change of spontaneous calcium signals (dF/F) of SF-1 neurons before and after mice entering central area, regardless of control or anxiety groups (unpaired Student's *t*-test, control, $P = 0.6626$; anxiety, $P = 0.9999$). (**d**) Schematic of photometry recording during the exploration in elevated plus maze (EPM). (**e**) Heat maps and (**f**) statistics analysis suggested that there is no significant change of spontaneous calcium signals (dF/F) of SF-1 neurons before and after mice entering open arms in both group (unpaired Student's *t*-test, control, $P = 0.7844$; anxiety, $P = 0.2520$). Data are means ± SEM, * $P < 0.05$.

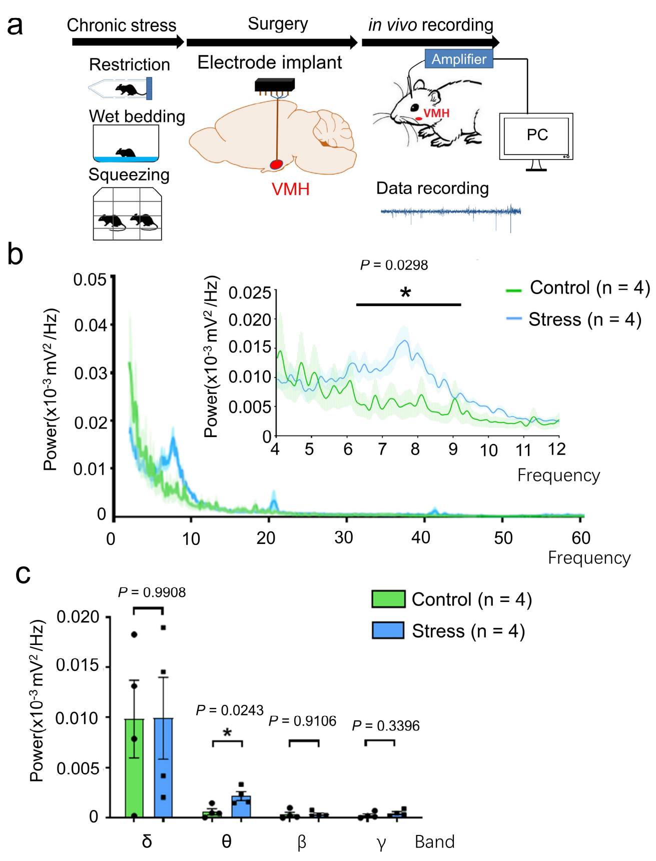

Supplementary Figure 2. *In vivo* electrophysiology recording of dmVMH neurons in control and stressed mice.

(**a**) Schematic of *in vivo* electrophysiology recording of dmVMH neurons in mice of control and stress group and related experimental strategy. (**b** and **c**) Power spectral density of local field potential (LFP) in dmVMH after chronic stress, with significant power improvement observed in theta band (two-way ANOVA, $P = 0.0298$, $F(1, 14) = 5.845$; unpaired Student's *t*-test, $P = 0.0243$, n = 4). Data are means ± SEM, * $P < 0.05$.

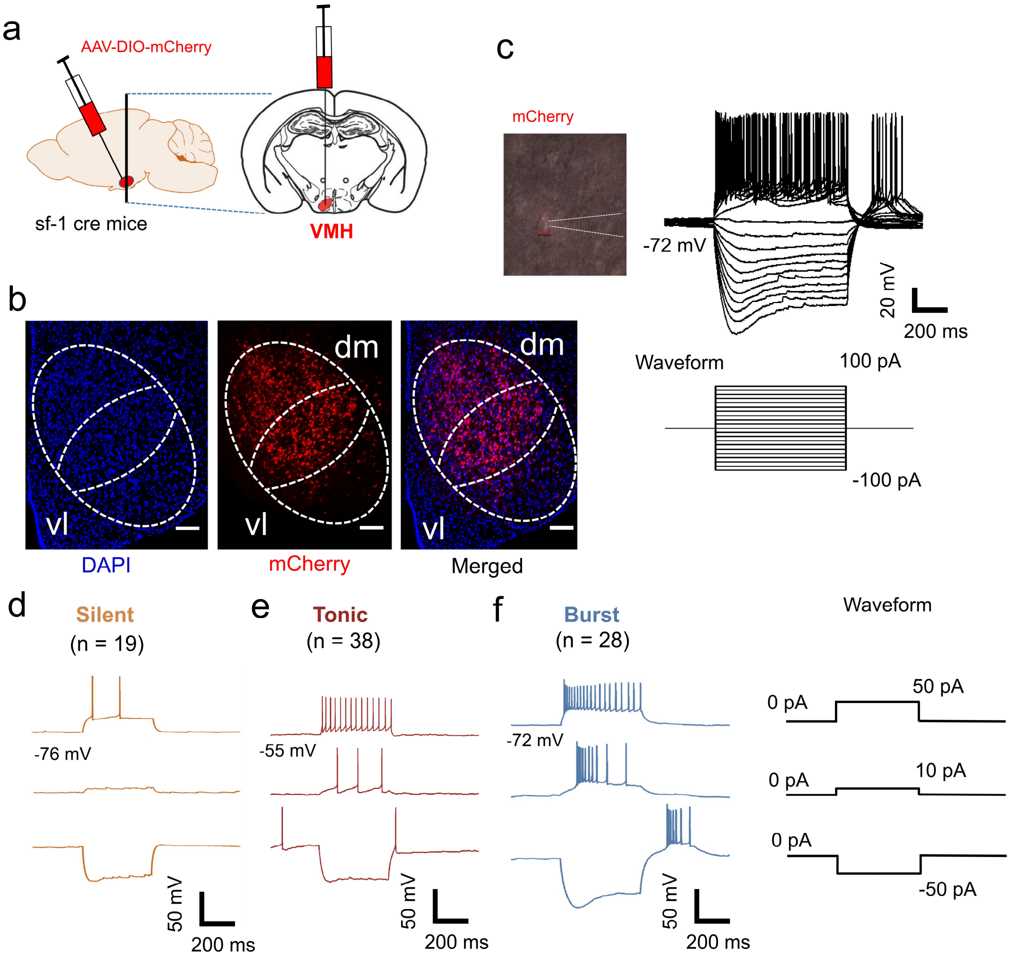

Supplementary Figure 3. Electrophysiological properties of dmVMH neurons subtypes.

(**a** and **b**) Schematic of location of dmVMH in coronal section slice of mouse brain, mCherry was specifically expressed in SF-1 (specific dmVMH marker) neurons. Scale bar is 300 μm. **(c)** Whole-cell recording trace from a dmVMH neuron, with current injection of -100 pA to 100 pA and 10 pA/step. (**d-f**) Representative traces of whole-cell recordings showing electrophysiological properties of silent (n = 19), tonic-firing (n = 38), and bursting (n = 28) neuronal subtypes in dmVMH. Three subtypes exhibit different electrophysiological activity at 50, 10, and -100 pA current injection.

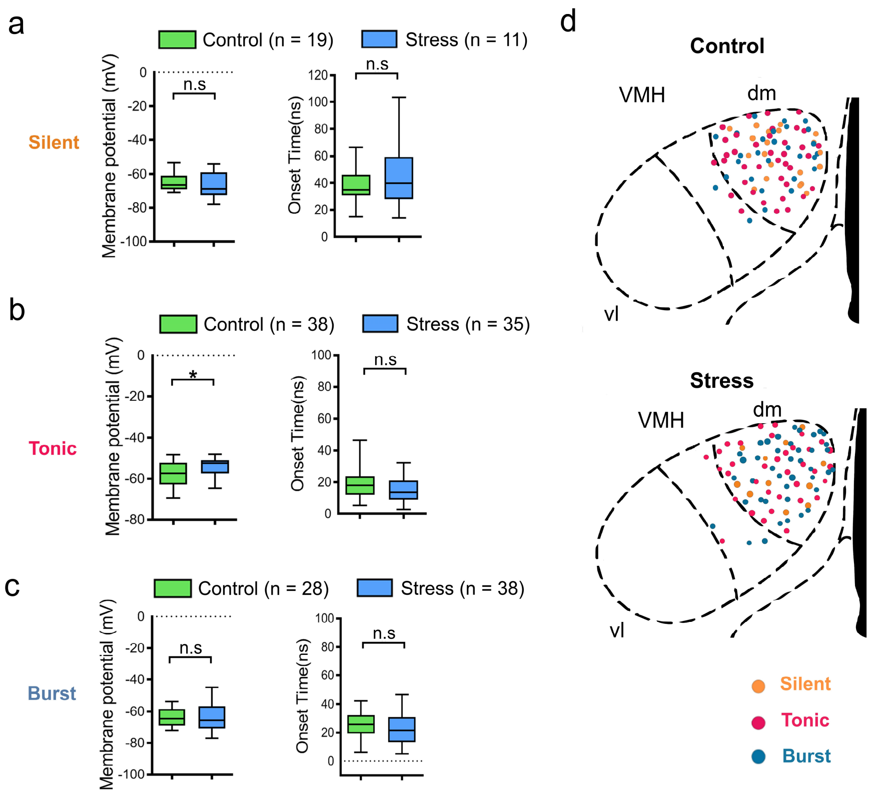

Supplementary Figure 4. Electrophysiological comparison of three dmVMH neuronal subtypes in control and stress groups.

(a) Resting membrane potential (RMP) and onset time of silent neurons in control (19 cells) and stressed mice (11 cells) exhibit no obvious difference (unpaired Student's *t*-test, RMP: *P* = 0.5549; Onset time: *P* = 0.3029). **(b)** RMP of tonic firing neurons in control (38 cells) are higher than that of stressed mice (35 cells), while no obvious difference observed in onset time (unpaired Student's *t*-test, RMP: *P* = 0.0047; Onset time: *P* = 0.0767). **(c)** No obvious difference has been found in RMP and onset time of burst firing neurons in control (28 cells) and chronically stress mice (38 cells) (unpaired Student's *t*-test, RMP: *P* = 0.9452; Onset time: *P* = 0.7704). **(d)** Location of each recorded neuron in dmVMH of control and stress group, no region specificity was

observed among three subtypes. Data are means ± SEM; $^{*}P < 0.05$, $^{**}P < 0.01$. The box plotted at the median extending from the 25-75th percentile, and the whisker represents Min to Max distribution.

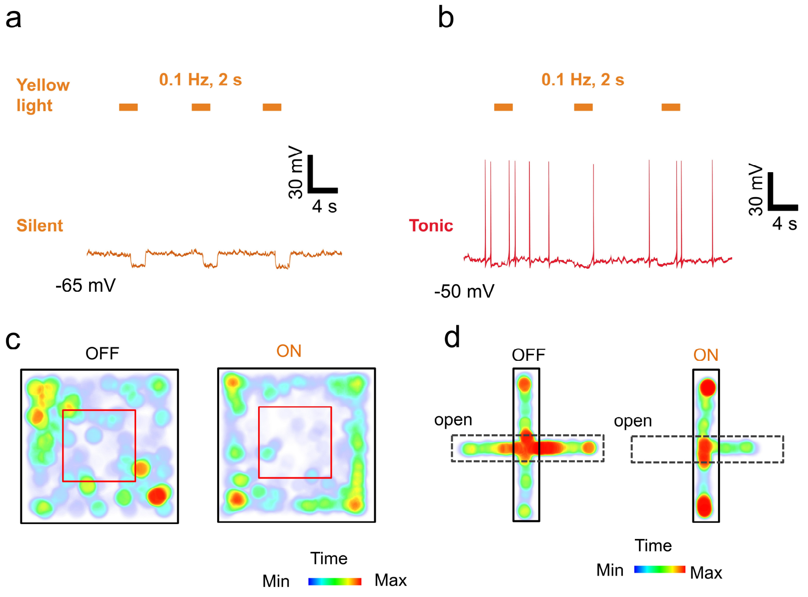

Supplementary Figure 5. Optogenetic manipulation of VMH SF-1 neurons and behavioral changes.

(a and **b)** Yellow light illumination (0.1 Hz) exerted no significant influence on silent or tonic-firing dmVMH neurons. **(c** and **d)** Optogenetically induced burst firing in VMH caused anxiety-like behavior in mice. Residence time in each site of open field before and during light illumination (blue, less time; red, more time); Residence time in each site of elevated plus-maze before and during light illumination (blue, less time; red, more time).

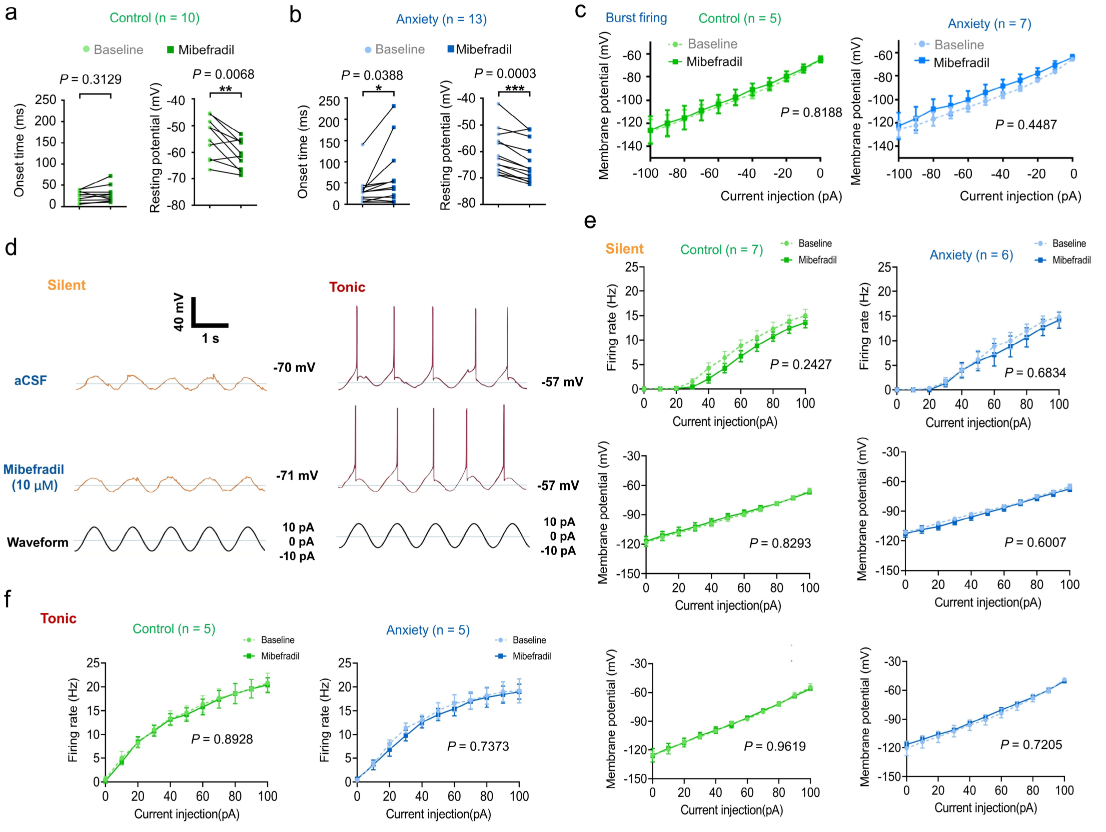

Supplementary Figure 6. Effects of mibefradil on electrophysiology of VMH neurons in control and anxiety group.

(a and **b)** onset time and RMP of dmVMH neurons from wild-type (n = 10) and anxiety groups (n = 13). Significant differences were observed in both onset time and RMP (paired Student's *t*-test, $P = 0.0388$ and $P = 0.0003$) in the anxiety group, whereas the control group only demonstrated obvious changes in RMP (paired Student's *t*-test, onset time, $P = 0.3129$; RMP: $P = 0.0068$). **(c)** Mibefradil application exerted no obvious influence on current-voltage curve of burst neurons (two-way ANOVA, control, n = 5 , $P = 0.8188$, $F(1, 10) = 0.0573$; anxiety, n = 7, $P = 0.4487$, $F(1, 12) = 0.6218$,). **(d)** Cosine current injecting into silent or tonic firing neurons failed to induced bursting. **(e)** No obvious differences were observed in frequency-current curve (two-way ANOVA,

control, P = 0.2427, F (1, 12) = 1.510; anxiety, P = 0.6834, F (1, 10) = 0.1763 with Bonferroni correction) and subthreshold membrane potential (two-way ANOVA, control, P = 0.8293, F (1, 12) = 0.1227; anxiety, P = 0.6007, F (1, 10) = 0.3770) of dmVMH silent neurons of control (n = 7 cells from 4 mice) and anxiety groups (n = 6 cells from 4 mice) after application of mibefradil. **(f)** Mibefradil exerted no obvious affect on the frequency-current curve (two-way ANOVA, control, P = 0.8928, F (1, 8) = 0.0193; anxiety, P = 0.7373, F (1, 8) = 0.1206) and subthreshold membrane potential (two-way ANOVA, control, P = 0.9619, F (1, 8) = 0.0025; anxiety, P = 0.7205, F (1, 8) = 0.1374) of dmVMH tonic firing neurons of control (n = 5 cells from 3 mice) and anxiety groups (n = 5 cells from 3 mice). Data are means ± SEM, two-way ANOVA performed with Bonferroni correction, * P < 0.05, ** P < 0.01, *** P < 0.001.

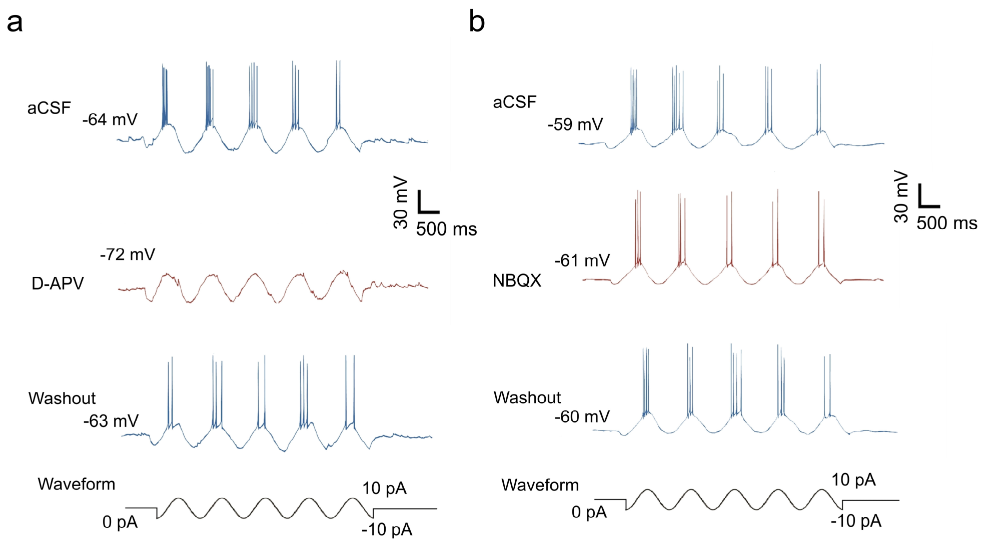

Supplementary Figure 7. Glutamate receptor is critical for generation of burst firing in dmVMH.

(a) Application of D-AP5 blocked generation of burst firing induced by cosine waveform current injection (-10 pA–10 pA, n = 5 cells). **(b)** Application of NBQX did not abolish generation of burst firing (n = 5 cells).

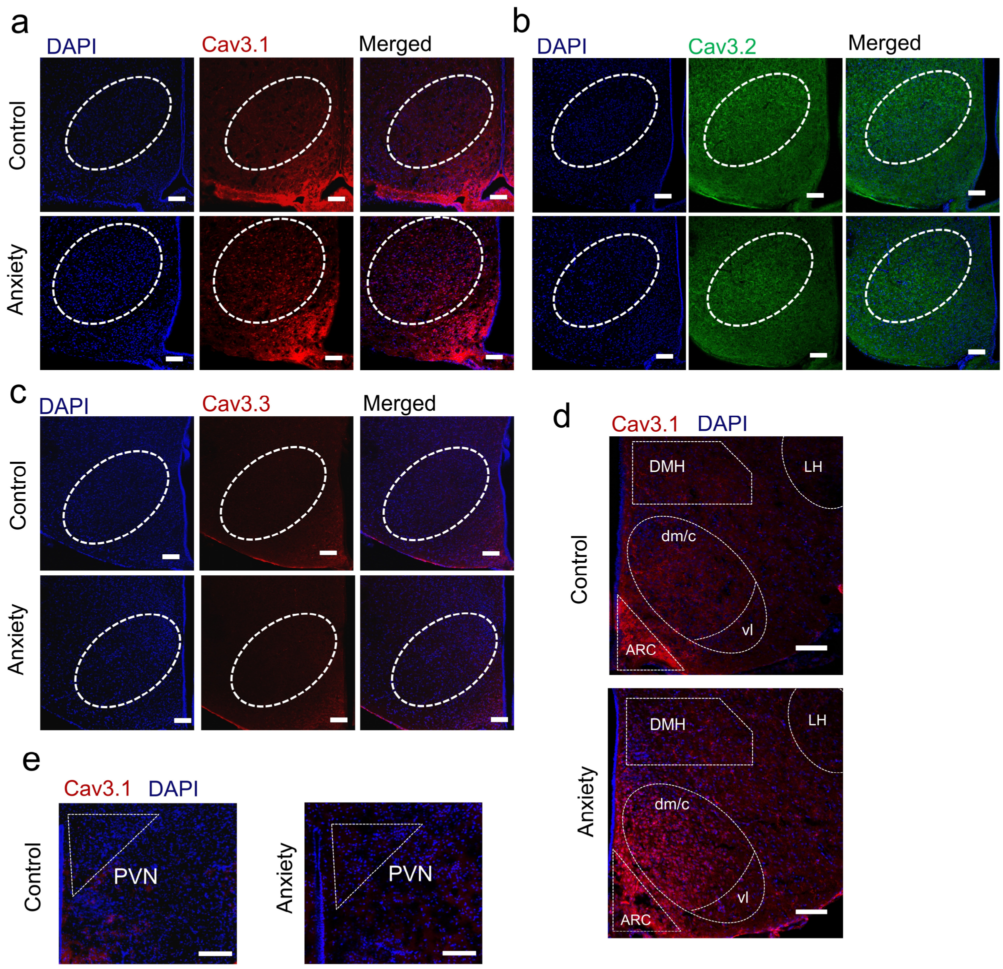

Supplementary Figure 8. Immunostaining of three T-VGCC in VMH and other hypothalamic regions. **(a)** Representative images of VMH Cav 3.1 immunostaining in mice of control and anxiety group. Slices from anxious mice exhibit higher Cav3.1 expression compared with the control mice. **(b)** Cav 3.2 and **(c)** Cav 3.3 immunostaining demonstrated no obvious difference between control and anxiety group. **(d)** no obvious changes of Cav3.1 signals were observed in other hypothalamic regions, including Arc, dorsomedial part (DMH), lateral part (LH) and paraventricular nucleus (PVN), between control and anxiety groups. Scale bar: 100 μm

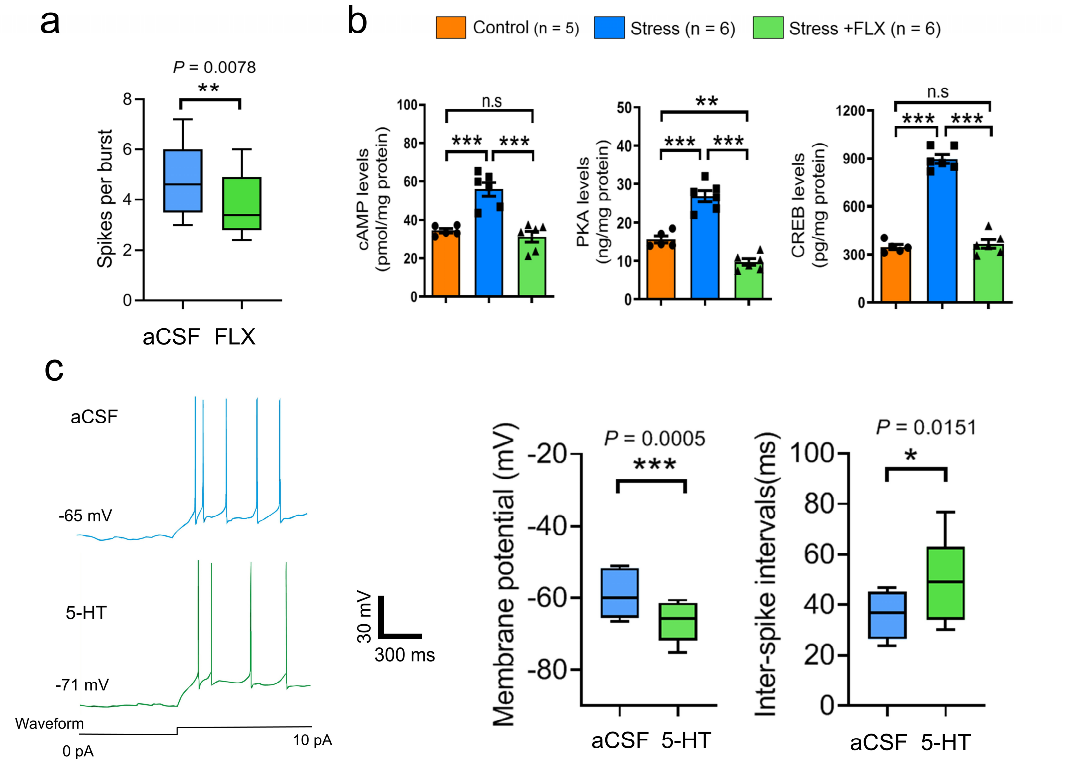

Supplementary Figure 9. Effect of Fluoxetine on the activity and cAMP-PKA signaling of dmVMH neurons.

(a) Application of fluoxetine (FLX) suppressed burst firing induced by cosine waveform current injection (-10 pA–10 pA, n = 5 cells from 4 mice, paired Student's *t*-test, *P* = 0.0078). **(b)** Application of FLX inhibited the enhanced cAMP-PKA signals induced by chronic stress. One way ANOVA with Bonferroni correction, cAMP: *P* < 0.0001; WT (n = 5 mice) versus stress group (n = 6 mice), *P* = 0.0004; stress versus stress + FLX group (n = 6 mice), *P* < 0.0001; WT versus stress + FLX group, *P* > 0.9999. PKA: *P* < 0.0001; WT versus stress group, *P* < 0.0001; stress versus stress + FLX group, *P* < 0.0001; WT versus stress + FLX group, *P* = 0.0091. CREB: *P* < 0.0001; WT versus stress group, *P* < 0.0001; stress versus stress + FLX group, *P* < 0.0001; WT

versus stress + FLX group, *P* > 0.9999. **(C)** 5-HT hyperpolarized dmVMH neurons and increased the ISI of burst firing neurons (paired Student's *t*-test, n = 6 cells from 4 mice, membrane potential, *P* = 0.0005; ISI, *P* = 0.0151). The box plotted at the median extending from the 25-75th percentile, and the whisker represents Min to Max distribution. Data are means ± SEM, * *P* < 0.05, ** *P* < 0.01, *** *P* < 0.001.
